# Supplementary material for: A protocol paper for the MOTION Study—A longitudinal study in a cohort aged 60 years and older to obtain mechanistic knowledge of the role of the gut microbiome during normal healthy ageing in order to develop strategies that will improve lifelong health and wellbeing
Source: PLoS One. 2022 Nov 18;17(11):e0276118. doi: 10.1371/journal.pone.0276118 (PMC9674124; doi:10.1371/journal.pone.0276118)
Supplement: S1 File — (PDF) [file pone.0276118.s001.pdf]

Study Title: **The MOTION Study – Microbiome of the ageing gut and its effect on human gut health and cognition.**

A longitudinal study in a cohort aged 60 years and older to obtain mechanistic knowledge of the role of the gut microbiome during normal healthy ageing in order to develop strategies that will improve lifelong health and wellbeing.

Annex 1: **MOTION Study Protocol**

IRAS Number: 241617

Date and Version No: Version 4 10.01.2019

Chief Investigator: Professor Simon Carding
simon.carding@quadram.ac.uk

QIB Investigator: Shelina Rajan shelina.rajan@quadram.ac.uk

NHS Investigators: Dr Simon Rushbrook simon.rushbrook@nnuh.nhs.uk
Dr Janak Saada janak.saada@nnuh.nhs.uk
Dr Ben Burton ben.burton@jpaget.nhs.uk

UEA Investigator: Professor Michael Hornberger
m.hornberger@uea.ac.uk

Norfolk and Norwich University Hospitals NHS Foundation Trust, James Paget University Hospitals NHS Foundation Trust and Norfolk and Suffolk NHS Foundation Trust – working in partnership.

TABLE OF CONTENTS

ABBREVIATIONS 10

1 BACKGROUND AND RATIONALE 11

1.1 Background 11

1.2 Rationale 15

1.3 Impact and Importance 15

2 AIM OF STUDY AND STUDY OBJECTIVES 16

2.1 Aim of Study 16

2.1.1 Primary Objective 16

2.1.2 Secondary Objective 16

3 OUTCOME MEASURES 17

4 STUDY TEAM 18

5 STUDY DESIGN 18

5.1 Cohorts 20

5.2 Subgroups 22

5.2.1 Subgroup 1 – Colonoscopy biopsies. 22

5.2.2 Subgroup 2 – Brain Magnetic Resonance Imaging (MRI) Scans 24

5.3 Study Delivery 25

6 RECRUITMENT 25

7 PARTICIPANT IDENTIFICATION 26

7.1 Stream 1 Participant Identification 26

7.2 Stream 2 Participant Identification 27

7.3 Stream 3 Participant Identification. 28

7.4 Inclusion Criteria 30

7.5 Exclusion Criteria 31

8 Phase 1 - Screening and Recruitment Phase 32

8.1 Telephone Contact 32

8.2 Pre-Study Visit 34

9 Phase 2 - Sample and Data Collection Phase 35
9.1 Study Visits 36
9.1.1 Baseline Visit (approximately up to 3 weeks from Pre-Study Visit). 36
9.1.2 Study Visits 1, 3, 5 & 7 39
9.1.3 Study Visits 2, 4, 6 & 8 40
9.1.4 Time windows for Study Visits 41
10 Incidental Findings 42
10.1 Cognitive Tests 42
10.2 MRI Scans 43
10.3 OCT/OCT (A) Scans 43
10.4 Full Blood Count, Biochemistry and Troponin C blood results 43
10.5 Colonoscopy Biopsies 43
11 STUDY PROCEDURES 43
11.1 INFORMED CONSENT 43
11.2 Informed Consent for Biorepository Storage 44
11.3 Study Specific Tests 44
11.3.1 Cognitive Tests 44
11.3.2 Optical Coherence Tomography (OCT) Scans 47
11.3.3 Optical Coherence Tomography Angiography OCT (A) Scans 47
11.4 Brain Magnetic Resonance Imaging (MRI) Scans. 50
11.4.1 MRI Analysis 51
11.5 Blood sampling 52
11.6 Health Questionnaires. 52
11.7 Physical Measurement Collection (Height, Tanita Scales Measurement, BP (blood pressure) and Hand Grip Strength Measurements). 52
11.8 Drop-off & collection of the stool collection pack. 53
11.8.1 Contents of stool collection pack 53
12 SCREENING AND ELIGIBILITY ASSESSMENT 53
13 SAMPLE HANDLING 54
13.1 Collection, Processing, Laboratory Analysis & Storage 54

13.1.1 Colon tissue Biopsy 54
13.1.2 Stool sample 56
13.1.3 Blood Sample 57
14 SAMPLE STORAGE 58
14.1 Long-Term Sample Storage 59
15 DATA COLLECTION 59
15.1 Cognitive Tests and Associated Questionnaires 59
15.2 Questionnaires 60
15.2.1 Full Health Questionnaire 61
15.2.2 Partial Health Questionnaire 61
15.3 Electronic Frailty Index 61
15.4 Physical Measurements 62
15.4.1 Height and weight measurement to calculate Body Max Index (BMI) 62
15.4.2 Body composition measurements 62
15.4.3 Blood Pressure (BP) 62
15.4.4 Hand grip strength measurement 62
16 EARLY TERMINATION/DISCONTINUATION/WITHDRAWAL OF PARTICIPANTS FROM STUDY 63
17 DEFINITION OF END OF STUDY 63
18 DEFINITION OF SERIOUS ADVERSE EVENTS 63
18.1 Reporting Procedures for Serious Adverse Events 64
19 STATISTICS AND ANALYSIS 64
19.1 Description of Statistical Methods 64
19.2 The Number of Participants 67
20 STUDY MANAGEMENT PLAN 68
20.1 Trial Management Group (Study team members) 68
20.2 Trial Management Oversight Group (Non- study team members) 68
21 DATA MANAGEMENT PLAN 69
21.1 Description of the data 69
21.2 Specific management of personal data 70

21.3 Specific management of samples: 70
21.4 Data collection/generation 71
21.5 Data sharing and access 71
21.6 Relevant institutional policies on data sharing and data security 72
21.7 Data Recording and Record Keeping 72
21.7.1 Data Retrieval & Storage 72
22 QUALITY ASSURANCE PROCEDURES 72
23 ETHICAL AND REGULATORY CONSIDERATIONS 73
23.1 Declaration of Helsinki 73
23.2 Approvals 73
23.3 Reporting 73
23.4 Participant Confidentiality 73
23.4.1 Use of audio/video recordings and anonymised quotes 74
. ... 74
23.5 Participant Identifiable Data Received Electronically 75
23.6 Expenses and Benefits 75
23.7 Other Ethical Considerations 75
23.7.1 Vulnerable Participants 75
24 FINANCE AND INSURANCE 76
24.1 Funding 76
24.2 Insurance 76
25 PUBLICATION POLICY 76
26 CORE TEAM MEMBERS AND EXPERTISE 76
27 Schedule of Events (Annex A) 78
28 REFERENCES 79

SYNOPSIS

To improve our understanding of the links between gut microbial populations and declining health in old age.

**Study Title**	Microbiome of the ageing gut and its effect on human gut health and cognition.	
**Internal ref. no. / short title**	The MOTION Study.	
**Study Design**	Longitudinal Prospective Cohort Study.	
**Study Participants**	Adult males and females aged 60 years and older.	
**Planned Sample Size**	360.	
**Planned Study Period**	Approximately 49 months.	
	**Objectives**	**Outcome Measures**
**Primary**	To describe the overall composition of the gut microbiota during ageing in a cohort of older individuals without existing serious health conditions on study entry.	Microbiota profiles based upon culturing isolates and/or DNA/RNA sequencing of serial samples collected from study participants.
**Secondary**	1. Establishment of data and a sample repository to facilitate future research in to ageing.<br><br>2. To estimate how gut microbe populations are associated with aspects of declining health including the gut, brain, immune and eye function and frailty.	Participant recruitment completed. Biobank of samples established within the Norwich Biorepository.<br><br>Gut: The gut and altered physiology assessed using various experimental protocols performed on colonic biopsy samples obtained during the course of the study.

		Brain: The brain and cognitive impairment measured using a battery of cognitive tests at start of study (t=0) and at 6-monthly intervals up to 48 months.<br><br>Immune System: Immonocompetency determined by altered immune cell repertoires and effector function assessed using blood sampling at regular (6 month) intervals using various experimental protocols.<br><br>Eye: Structural changes including retinal nerve fibre layer thinning assessed by retinal scanning (OCT) at the start, mid-point and end of the study.<br><br>Metabolism: Metabolism and metabolic health assessed using faecal and blood samples obtained at regular (6-12 month) intervals.
	3: To determine if changes in the gut microbiota detected over time equally affect its different members?	Identifying change in virus (DNA/RNA), archaea and fungal populations from faecal samples obtained at regular intervals (6 months) throughout the study.

ABBREVIATIONS

BCSP	Bowel Cancer Screening Programme
CSP	Colonoscopy Surveillance Programme
DC CRF	Data Collection Case Report Form
eFI	electronic Frailty Index
FOBt	Faecal Occult Blood test
GCP	Good Clinical Practice
GLP	Good Laboratory Practice
GP	General Practitioner
HRA	Health Research Authority
ICF	Informed Consent Form
MCI	Mild Cognitive Impairment
NHS	National Health Service
NNUH	Norfolk & Norwich University Hospital NHS Foundation Trust
NRES	National Research Ethics Service
NSFT	Norfolk and Suffolk NHS Foundation Trust
OCT(A)	Optical Coherence Tomography (Angiography)
PI	Principal Investigator
PIC	Participant Identification Centre
PIL	Participant Invitation Letter
PIS	Participant Information Sheet
QIB	Quadram Institute Bioscience
QI CRF	Quadram Institute Clinical Research Facility
QIE	Quadram Institute Endoscopy
R&D	NHS Trust R&D Department
REC	Research Ethics Committee
SOP	Standard Operating Procedure
UEA	University of East Anglia

1 BACKGROUND AND RATIONALE

1.1 Background

Humans have co-evolved with populations of colonising microbes and their genomes to establish a mutually beneficial relationship (Figure 1). The gut microbiome represents the largest population of resident microbes in humans and is highly diverse and plays essential roles in digestion and pathogen protection [1]. Perturbations in populations of gut bacteria (dysbiosis or dysbacteriosis) in response to, for example, infections or drugs, have been associated with an increased risk of various chronic diseases including obesity, cancer and inflammatory bowel disease (IBD) [1, 2] (Figure 1).

In older individuals an array of complex and characteristic clinical changes that includes a basal proinflammatory state (so called "inflammaging"), can directly interface with gut microbes of older adults that can enhance susceptibility to diseases accompanying aging, and reduce healthy lifespan [3, 4]. Studies in older adults demonstrate that the makeup and functional attributes of the intestinal microbiota correlates with lifestyle (e.g. diet and location of residence) and behaviour (e.g. medications), and basal level of inflammation. Links exist between the intestinal microbiota and a variety of clinical problems plaguing older adults, including physical frailty, *Clostridium difficile* colitis, vulvovaginal atrophy, colorectal carcinoma, and atherosclerotic disease. These links are however, principally associative with very little evidence of causality [5].

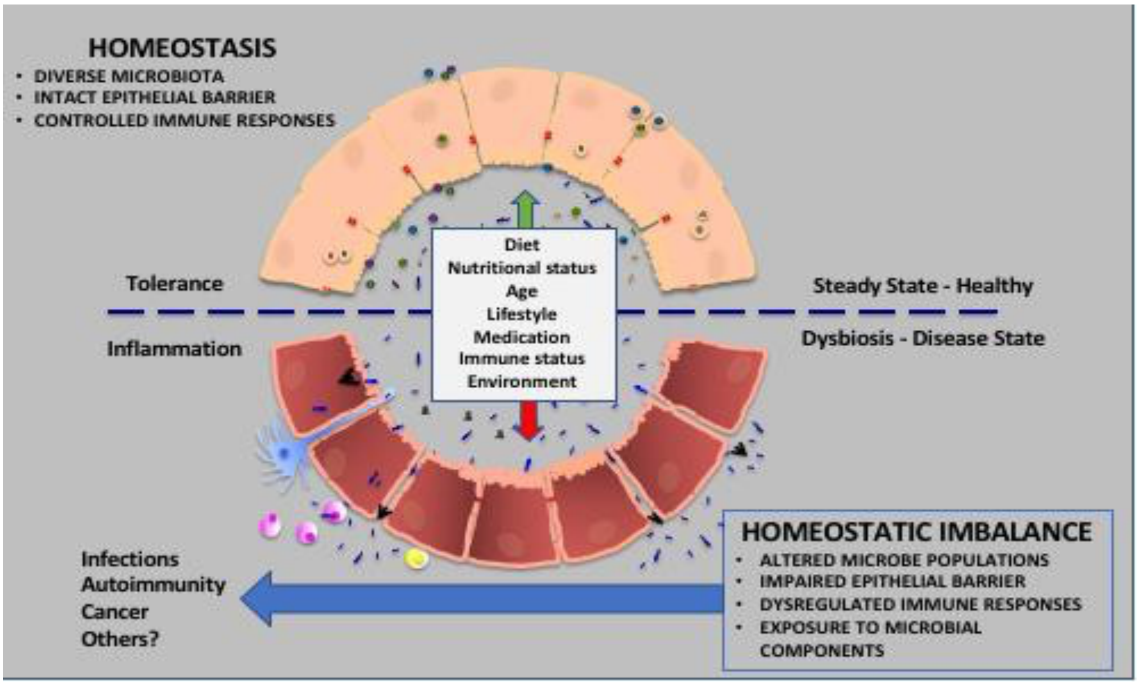

Figure 1: The role of the gut microbiome in health and dysbiosis.

The gut microbiome establishes a mutualistic relationship with its host and contributes to maintaining homeostasis. Its composition can be influenced and changed by numerous host (e.g. genome, age), environmental (e.g. diet, medicines, infections), and lifestyle and behavioural factors (e.g. travel, habitat). Changes in the structure and/or function of the microbiome (dysbiosis) can result in damage to, or infection of, barrier epithelial cells enabling microbes and microbial products to cross the compromised barrier and gain access to underlying tissues and immune cells, leading to immune activation and (chronic) inflammation (adapted from [6]).

Age is a major risk factor for the development of cognitive dysfunction with dementia being one of the most common disorders linked to ageing [7]. Dementia affects an estimated 47 million people worldwide and is projected to affect over 131 million people by 2050 [8]. Cognitive function declines with age, ranging from relatively minor everyday slips of action, through subjective cognitive decline, mild cognitive impairment (MCI), then to major or mild neurocognitive disorder/dementia in some instances. Up to 50% of those with MCI are predicted to develop dementia within 5 years [9].

The development of new treatments to prevent dementia is hindered by a lack of predictive biomarkers. The gut microbiome is a potential marker for progressive declining intrinsic capacity, which is a composite of all the physical and mental attributes on which an individual can draw, not only in older age but across their lives. Evidence of age-associated changes in the gut microbiome comes from population-based studies, one of the largest and most comprehensive was ELDERMET, a study that examined the faecal microbiome of elderly individuals living in the community or in care homes in Ireland [10]. The results identified differences in the composition of the gut microbiome of elderly individuals compared with younger individuals, and that these differences were associated with poorer health status in the elderly (e.g. poor dentition, dysphagia, loss of salivation, decreased physical activity and constipation).
However, in relying on data from samples taken at a single timepoint, this study provides only a 'snapshot' of what is happening but little insight into the dynamics or stability of any changes in the gut microbiome over time in different individuals or groups of participants. It is also impossible to determine whether the changes noted are drivers or consequences of unhealthy ageing. In addition, this study did not include assessments of mental health and cognitive function, which are major comorbidities associated with ageing. The fact that structural changes within the gut microbiome occur during ageing is a consistent finding across different studies [11]. However, the scale and nature of the changes noted vary considerably between studies, and amongst individuals within the same study. Larger longitudinal studies with sequential (serial) sampling of the gut microbiome during ageing would provide a clearer picture of how the gut microbiome changes during ageing, and whether it is a contributing factor to declining health in old age and/or in the development of neurodegenerative disorders and dementia.

The link between potentially pathogenic changes in the gut microbiome (dysbiosis) in individuals with neurodegenerative disorders comes mainly from observational studies in patients with Alzheimer's (AD) or Parkinson's disease (PD), and from animal (rodent) models. Analysis of the faecal microbiome of dementia patients has identified an increased prevalence of *Prevotellaceae* and *Enterobacteriaceae* species in PD, and an increased prevalence of *Clostridia*, *Bacteroides* and *Verrucomicrobia* species in AD patients. compared with healthy individuals [12, 13]. In addition, many PD and AD patients reported gut symptoms including irritable bowel syndrome prior to neurological symptoms developing [14]. Furthermore, antibiotic treatment to eradicate gut pathogens (e.g. *H. pylori*) can improve L-dopa

action and reduce clinical symptoms in PD patients. Perhaps the most compelling evidence for a causal link between gut microbes and neurodegenerative disease comes from the finding that, when faecal microbiomes from PD patients are transferred into PD-susceptible mice, symptom development accelerates and the mice suffer greater motor dysfunction than control mice receiving faecal microbiomes of healthy individuals [15]. Although collectively these findings implicate the gut microbiome in the pathogenesis of neurodegenerative disorders, the studies carried out to date have failed to identify which species of bacteria (or any other member of the gut microbiome; viruses, archaea, fungi, protozoa) are critically important for mental health and in the development of dementia, when during ageing do changes in the population size and/or function of critical species occur, and once established how do these critical species influence brain signalling, neuroinflammation and pathology?

Another screening test with potential as a biomarker for ageing and dementia development is retinal morphometry. The retina is the only part of the central retinal nervous system that can be directly visualized. Optical Coherence Tomography (OCT) is a rapid, non-invasive imaging tool that can produce 3-dimensional cross-sectional images of the retina, and permits precise and accurate measurement of the thickness of individual retinal components [16]. The retinal nerve fiber layer (RNFL) is the innermost layer of the retina and is comprised of the retinal ganglion cell axons, which link the outer neuroretina to the dorsal lateral geniculate nucleus, where synaptic connections lead to the visual cortex. In a very recent multi-centre study of individuals aged between 40 and 69 years of age, OCT imaging revealed that a thinner RNFL was associated with worse cognitive function in individuals with no neurodegenerative disease; it was also associated with a greater likelihood of subsequent cognitive decline [17]. This makes a strong case for regarding retinal anatomical measures as a potentially useful screening marker to identify those at risk of developing dementia.

The studies carried out to date highlight the need to develop a more holistic and integrated understanding of human ageing: There is a need to understand how (or whether) changes in one organ system (e.g. the gut) are related to changes occurring elsewhere in the body (e.g. in the brain). In order to begin to answer these fundamentally important questions we have designed the MOTION study to obtain a clearer picture of how the gut microbiome changes during ageing in a cohort over the age of 60 years without existing serious medical conditions at the point of study

consent, and how these changes relate to declining intrinsic capacity and increasing cognitive impairment.

1.2 Rationale

We aim to determine how age-associated changes in the gut microbiome are associated with declining physical and mental capacity in a stable and well-defined East Anglian ageing population over the age of 60 years that are stratified according to their cognitive function and degree of cognitive impairment as detailed in Section 5. By analysing the intestinal microbiome of individuals in these groups of participants over a 4-year period and relating it to measures of their age-associated declining intrinsic capacity (i.e. measures of frailty and the function of different organ systems including the gut, brain, liver and eye) MOTION will allow us to establish a much clearer picture of how the gut microbiome impacts on healthy ageing. A comprehensive longitudinal multiparameter-based study such as this is novel and has not been previously undertaken.

1.3 Impact and Importance

The UK population is ageing although the average healthy life span is not extending at the same rate as total lifespan [18]. Advances in medicine and public health mean that people are living longer; alongside demographic drivers this results in an increasing proportion of older people in the UK population. However, a significant proportion of that increased lifespan is spent in a prolonged state of declining health and wellbeing [19]. Those in later life spend longer living with chronic medical conditions, reduced independence and in need of care - all of which places increasing pressure on medical, health and social services. There is a pressing social and economic need for research to promote health and independence into old age. The development of strategies that prevent or delay age-related disease and maintain lifelong health are a major goal over the coming decades. A key, achievable strategy for healthy ageing is to focus on the gut microbiome**,** which in view of it being increasingly linked either to health, or chronic disease makes it a promising target for strategies that specifically contribute to the health status of at-risk and vulnerable individuals.

2 AIM OF STUDY AND STUDY OBJECTIVES

2.1 Aim of Study

To improve our understanding of the links between gut microbial populations and declining health in old age.

2.1.1 Primary Objective

To describe and define the composition of the gut microbiome during ageing in a cohort of individuals (60 years of age and older) without existing serious health conditions.

Data from serial samples in an initially healthy cohort will be analysed to identify correlations between changes in the gut microbiome during ageing in three cohorts of individuals defined by different degrees of cognitive function. Associations will be drawn using statistical modelling of data obtained from the structural and functional analyses of faecal microbiome populations and mental (i.e. cognitive function) and physical (e.g. weight changes, blood pressure) health assessments. This will provide a global picture of later-life microbiome deterioration in relation to the health status of our ageing participants. Importantly, samples will also be used to answer more specific research questions using innovative experimental systems, tools and methodologies. This will include using *in vitro* and *in vivo* models to define how specific microbes (or their products which are known to provide health benefits) change during ageing in cohorts categorised according to their risk of developing dementia. The overarching aim is to provide key insights into how later-life microbiome profiles correlate with health outcomes, which will inform the development of new non-toxic, microbe-based therapies to promote and maintain health and/or treat disease.

2.1.2 Secondary Objective

1. Establishment of data and a sample repository to facilitate future research into ageing.

2. To estimate how gut microbe populations affect aspects of declining health including the gut, brain, immune and eye function and frailty.

3. To determine if changes in the gut microbiota detected over time equally affect its bacterial, viral and fungal constituents.

3 OUTCOME MEASURES

Objectives	Outcome Measures	Timepoint(s) of evaluation of this outcome measure (if applicable)
**<u>Primary Objective</u>**<br><br>To describe how the gut microbiota changes over time in a cohort of individuals aged 60 years and older without existing serious health conditions on study entry.	Profile of the structure and composition of the bacterial component of the gut microbiota in a cohort of ageing individuals without serious health conditions.	Samples collected at start of study (t=0) and at 6 monthly intervals up to 48 months with sample analysis ongoing throughout the study.
**<u>Secondary Objectives</u>**<br><br>1. Establishment of the study cohort and sample repository to facilitate future research into ageing.	Successful recruitment to all three study groups.<br>Obtain all samples from individual participants according to procurement schedule. Deposit samples in Biorepository.	Recruitment completed within 24 months of study start date.<br>Sample repository initiated from study start date with further collections at 6 monthly intervals up to study end point.
2. To link age-associated changes in the gut microbiota to other measures of declining	Profile of the structure and composition of the bacterial component of the gut microbiota in	Comparative analysis of microbiota profiles with data obtained from additional datasets that

health and (organ) functionality.	groups of participants stratified according to their cognitive function and risk of developing dementia.	inform on the functionality of the gut, brain, eye, immune system using samples obtained throughout the study.
3. Do changes in the gut microbiota detected over time equally affect its different members?	Profile of bacteria, virus, archaea and fungal populations.	Measured at 6-monthly time points.

4 STUDY TEAM

This study will run using a combination of research staff and clinical staff. The study is led by Professor Simon Carding, Gut Microbes & Health Programme Leader at the Quadram Institute Bioscience (QIB), in collaboration with clinicians at the Norfolk & Norwich University Hospital NHS Foundation Trust (NNUH) and researchers at the University of East Anglia (UEA). It is anticipated that this study will be adopted on to the NIHR research portfolio allowing support through dedicated research nursing staff to support the study (consent, data collection etc.). See Section 26 for full study team.

5 STUDY DESIGN

The MOTION Study is an approximately 49-month longitudinal prospective study of 360 participants aged 60 years and over.

Participants will not have any severe medical conditions at baseline. By severe medical conditions we mean those that are known or are suspected to significantly affect either the gut microbiome or cognitive function. The rationale for the exclusion of severe medical conditions is that we want to be able to attribute changes in the dynamics of aging, particularly cognitive aging, to differences in the intestinal microbiota, and to describe the normal variation in microbiota (to which pathological changes or those induced by medications might later be compared). The presence of a baseline condition that would affect cognitive function by an

alternative process, for example untreated depression, would add variance to this outcome measure and make estimation of our associations of interest more difficult. See section 7.5 for the list of exclusion criteria.

The sample will be a convenience sample, selected through three different streams that together aim to identify participants from across the cognitive spectrum from healthy to those with mild cognitive impairment, while also enabling the collection of gut biopsies from a significant number through the course of their routine clinical care. While this is not a random sample and so will not be strictly representative of the population, there is little reason to expect that any particular selection bias will occur with respect to our research objectives.

Participants will be recruited via three streams (Primary Care as Patient Identification Centres, the NHS Bowel Cancer Screening Programme {BCSP} and the Norfolk & Suffolk NHS Foundation Trust {NSFT}) within the first two years to collect questionnaire and assessment data as well as biological samples at various time points. The research study will take place at the Quadram Institute Clinical Research Facility (QI CRF) where study visits will take place and QIB laboratory facilities. Additional study visits will be undertaken at the NNUH (QI Endoscopy, Imaging department) and Beccles Hospital.

A summary of the MOTION study design is detailed in Figure 2 below.

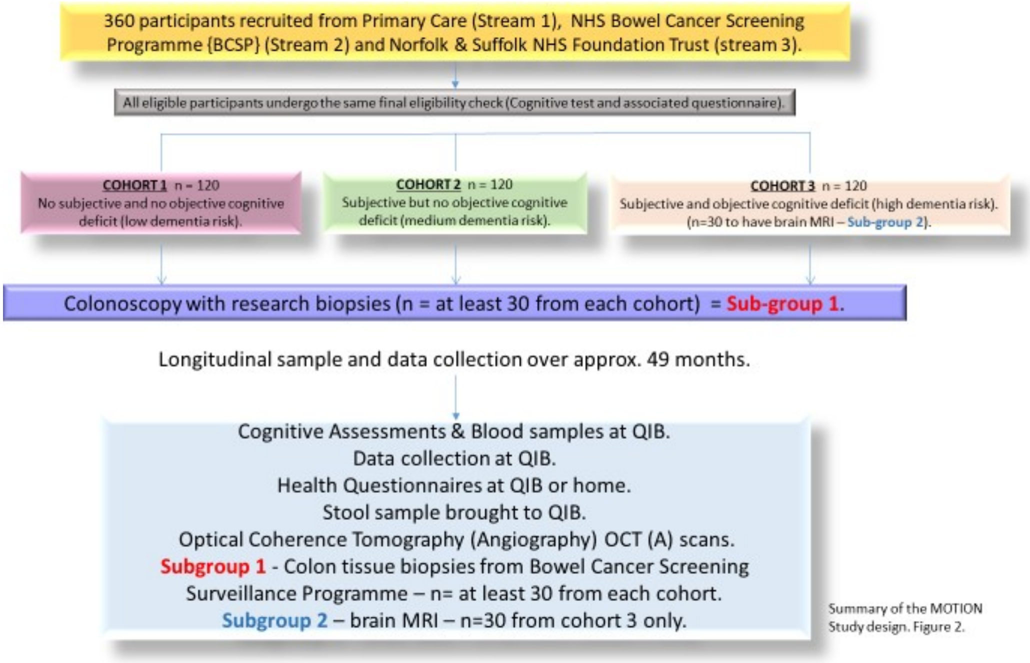

Summary of the MOTION Study design. Figure 2.

360 Study participants will be recruited using three streams: -

Stream 1 – Primary Care search of eligible participants.
Stream 2 – Utilisation of the Colonoscopy Surveillance Programme (CSP) waiting list which is part of the NHS BCSP for the purpose of recruiting to this study.
Stream 3 – NSFT clinical teams and Research Nurses.

At the Pre-Study Visit, all consented participants will undergo the following as a final eligibility check following the consenting procedure: -

- Mini Addenbrooke's Cognitive Examination (mini ACE) – a cognitive screening test.
- Cognitive Change Index (CCI) - self-completed measure of perception of the participant's cognitive decline.

For a full description of all 9 Cognitive Tests, the associated questionnaires and scoring thresholds that will be used in this study, please see Section 11.3.1. These will be delivered by trained Study Researchers/Study Research Nurses. Professor Michael Hornberger (UEA Collaborator and Co-Principal Investigator) with support from an experienced Study Researcher/Research Assistant will score each participant and assign them in to one of three cohorts detailed below.

5.1 Cohorts

120 participants will be recruited in to each of the 3 cohorts as below.

- Cohort 1 – No subjective and no objective cognition deficit (low risk of developing dementia).
- Cohort 2 – Subjective but no objective cognitive deficits (medium risk of developing dementia).
- Cohort 3 – Subjective and objective cognitive deficits (high risk of developing dementia) recruited primarily through NSFT specialist clinical teams.

We would like to stress that the results of the cognitive tests are not clinically relevant but are only important for the purpose of this research. For this reason, results of the cognitive tests and stratification to one of the three cohorts will not be discussed with recruited participants as we do not wish to cause any undue anxiety or concern. In addition, this may also impact negatively on the study in

terms of participants either withdrawing themselves or altering their dietary and/or lifestyle behaviour.
The NSFT provided feedback from the Norwich Older Person's Forum regarding withholding information about a cohort they might be assigned to in a clinical trial. The attendees had no ethical concerns about the categorisation of participants in to groups was not clinically meaningful as they understood that assessments used in a trial alone and in the absence of a specialist clinician would have no relevance.

It is anticipated that all three cohorts may be populated from more than one stream. At the time of taking Informed Consent from the participant, it would not be known by the Study Researcher team which cohort the participant will be in. In the event of a cohort reaching its maximum number of 120 participants, the participant will be withdrawn as their scoring did not meet a required threshold for study entry in to any of the cohorts (see Section 16).

Participants in cohort three will be populated with individuals with mild cognitive impairment (MCI) which refers to cognitive decline from a previous level of functioning. Both subjective and objective evidence are used to identify currently healthy ageing individuals who are at a high risk of developing dementia longitudinally [20]. Data from eleven studies carried out across six countries have suggested that MCI is a surprisingly common condition, especially in industrialized nations, affecting between 5 and 37% of the studied populations [21]. It is important to note however that MCI is not the same as a diagnosis of dementia because up to 40% of MCI patients revert back to health within 12 months [22]. We are confident that high, medium and low risk participants can be identified over the participant recruitment period of 2 years, even with a possibly higher dropout rate for the high and medium-risk subjects.

We expect to identify in excess of 3,500 potential participants with MCI (Norfolk and Clinical Commissioning Group prevalence figures provided by Tim Winters, Head Public Health Information, Norfolk County Council, April 2018) using objective cognitive deficits; from an annual conversion rate of 10% to dementia, this will make it possible to identify at least 120 high-risk participants within the recruitment period. This assumes that 50% of those meeting the eligibility requirements can potentially be recruited to the study. In assuming an annual attrition rate of ~20%, our conservative estimate is that the number of participants required can be recruited within 2 years.

It is expected that the study will be adopted on to the NIHR Portfolio and receive support for recruitment in Primary Care from CRN Eastern. QIB will collaborate with the NSFT whose specialist clinical teams conduct Memory Assessment Clinics for GP-referred patients. This will help enable recruitment of potential participants to cohort 3. The NSFT report that approximately 200 patients per week are referred by their GP for memory associated reasons. The MOTION Study will be promoted by the use of a study fliers (Annex 6.1) and posters (Annex 6.2) within these clinic settings which provide the Study Researcher's contact details. As a tool to promote mental health research, the NSFT clinical teams request their patients to complete a 'Consent to Research' form (Annex 12). This form provides explicit consent for the NSFT Research Team to contact them about any new research studies that may be of interest to them. The NSFT provides a wide range of health and social care services, specialising in mental health and wellbeing, and have a dedicated Research team to deliver clinical studies.

5.2 Subgroups

Additional sample and data collection will be carried out on two subgroups of participants which are detailed below:

5.2.1 Subgroup 1 – Colonoscopy biopsies.

Public Health England Screening leads the national population screening programmes, which are delivered by the NHS. These identify apparently healthy people who may be at increased risk of a disease or condition, enabling earlier treatment and better-informed decisions[23].

Under this NHS screening programme, all patients from the age of 55 years who are registered with a GP in Norfolk will be invited to attend the NHS routine clinical care Bowel Cancer Screening Programme (BCSP) and initially undergo either a flexible sigmoidoscopy or a postal FOBt (Faecal Occult Blood test). If either is positive, the patient will be invited for further investigation to have their routine first colonoscopy as part of the BCSP at the Norfolk & Norwich University Hospital (NNUH) Endoscopy Unit. The colonoscopy procedure is performed within the Endoscopy Unit by a specially trained endoscopist. This out-patient service is located at the QI.

The result of this first colonoscopy will determine if they have a repeat colonoscopy after 1 year, or after 3 years. The individuals on either of these waiting lists are termed as being on the Colonoscopy Surveillance Programme (CSP) of the BCSP. Participants who are recruited from Stream 2 will be from the NHS CSP. At the point of consenting for the study, participants from Streams 1 & 3 may not know if they will need to undergo a colonoscopy as part of the BCSP in the future. However, the study consent form will explicitly request that the participant informs the Research team if they receive an invitation from the BCSP team and that this team will be informed of every participant enrolled on to the study.

This subgroup will require at least 30 participants from each cohort to have colonic biopsies taken. Based upon previous and ongoing studies at QIB using human biopsy samples, this number of participant samples is sufficient to provide the required number for various downstream analyses and production of robust data sets to establish structural and functional profiles of tissue-associated microbial and host cell populations. This will enable us to formulate testable hypotheses to undertake future mechanism-based research. Over the period 01/01/2019 to 31/12/2021 the NNUH-QI Endoscopy department is scheduled to carry out bowel cancer surveillance colonoscopy procedures on approximately 1,400 patients from which we would recruit participants to this subgroup. We are confident therefore in obtaining the required number of biopsies for our study. See Section 7.2.

Participants from Stream 2 will be having a routine care surveillance colonoscopy which may or may not include the need for routine clinical biopsies to be taken. Participants in this study will have, in addition to any routine clinical care biopsies, and when possible 6-8 pinch biopsies each measuring $3mm^3$, taken from the large bowel for research purposes. If any routine biopsies need to be taken as part of the CSP clinical care, research biopsies will only be taken after the routine care biopsies have been taken. It is acknowledged that it may not be possible to achieve the minimum of 6 biopsies in some cases. Consent for the routine care colonoscopy and the procedure will be performed by Dr Simon Rushbrook or a member of his team.

For participants enrolled on to the study from Stream 2, the design of the CSP lends itself for potentially obtaining a second set of colonoscopy biopsies (provided the participant is in agreement). This is possible because the participant will be placed in the 1 year or 3 year waiting list for their next colonoscopy and is dependent on clinical findings from the first BCSP colonoscopy (study duration from the Baseline

visit is 48 months). It is acknowledged that some participants may have completed several Study Visits or are nearing the end of the study by the time they have undergone a colonoscopy as part of the BCSP.

5.2.2 Subgroup 2 – Brain Magnetic Resonance Imaging (MRI) Scans.

30 participants from the high-risk cohort will form subgroup 2 and will undergo MRI-based brain imaging on two occasions (at the beginning and end of the study) at the NNUH Imaging Department. Imaging protocols will adopt the same design and concept as the Alzheimer's Disease Neuroimaging Initiative 2 (ADNI2) protocol (ClinicalTrials.gov Identifier: NCT01231971) to measure the progression of MCI (1h total scanning time). The sequence protocol for the MRI will involve structural and functional sequences, which are established on the NNUH MRI scanner by Professor Hornberger and commonly used in his dementia cohorts. Structural sequences will allow measurement of grey and white matter integrity in participants, whereas functional sequences will allow measurement of brain connectivity and perfusion changes. We hypothesize that medial temporal structures will be intact in low risk participants but those in the medium risk group might already show subtle structural and functional changes.

MCI can be a very emotive subject and the researchers do not wish to cause participants any undue stress or anxiety. For this reason, participants will not be made aware of the 3 cohorts, but will be told that out of 360 participants, 30 will be offered a brain MRI scan based on the result of their Pre-Study Visit test results (mini-ACE and CCI). They will also be reassured that the test result, in combination with being offered the MRI scan, is not indicative of any clinical diagnosis and is purely for the purposes of this research study. The brain MRI scans are optional and does not affect participation in the study.

Each consecutive participant who is enrolled in to cohort 3 will be offered the brain MRI scan. If the participant declines this or is not medically able to have the scan, it will be offered to the next consecutive participant until the required number of 30 is achieved. This group will form subgroup 2.

At the point of consenting at the Pre-Study Visit, the Study Researcher will not know which cohort the participant will be placed in and will therefore ascertain eligibility and personal preference for imaging for all enrolled participants for consistency by using the Pre-Study Visit Check-list (Annex 9).

The 30 participants who form subgroup 2 (as described in Section 5.2.2) will have their brain MRI scan performed within 6 weeks of the Baseline Visit appointment and up to 6 weeks prior to the final study appointment (Study Visit 8). MRI appointment letters (Annex 24) and the NHS MRI Safety Questionnaire (Annex 17) will be posted to the participant by the Study Researcher.

5.3 Study Delivery

The study will be delivered by Study Researchers (specifically trained and experienced members of QIB staff). Externally, experienced Clinical Research Network (CRN) Research Nurses based within NNUH and NSFT Research team will also be involved in study support and delivery.
All staff involved in the study who are external to QIB will be trained in every aspect of study delivery by the QIB Study Researchers.

6 RECRUITMENT

To promote awareness of the study and to maximise recruitment potential, the following organisations have been approached:

❖ CRN Eastern will supply research active GP sites with study fliers and posters for display. Fliers will be formatted for upload on to TV screens in patient waiting areas.

❖ Join Dementia Research (JDR) [24]. To facilitate the ongoing recruitment to their existing portfolio of research studies, the NSFT Research Team have forged strong links with JDR. The NSFT Research Team and JDR will work jointly to search their database of potentially eligible participants, both MCI and non-MCI. JDR have also agreed to advertise the study using the MOTION Study Press Release (Annex 6.3).

❖ NSFT Insight Magazine [25] will feature the Motion Study Press Release.

❖ Healthwatch Norfolk [26] is the consumer champion for health and social care in the county have agreed to publicise the MOTION Study Press Release regularly in their newsletter.

❖ NNUH Pulse magazine will also feature the MOTION Study Press Release as a rolling article.

❖ In order to maximise the recruitment potential, the study will also be advertised using Social Media and through the medium of local radio, television and press articles, for example Norfolk Magazine.

❖ Participants can view the Full PIS and complete and online reply form www.quadram.ac.uk/motionstudy

❖ Study fliers and posters will be displayed in communal areas of the QIB so that visitors can access these.

❖ Any appropriate independent or NHS organisation willing to use ethically approved documentation to promote the MOTION Study.

7 PARTICIPANT IDENTIFICATION

The study is looking to recruit 360 eligible participants aged 60 years and above. This will be achieved by adopting a strategy of using 3 separate recruitment streams.

Once participants have either been identified as eligible or expressed an interest in receiving the study information, they will be sent a Participant Invitation Letter (PIL, Annex 2), a Summary Participant Information Sheet (PIS, Annex 4) and a Full PIS (Annex 5). Every Full PIS that is sent out will have a reply sheet attached for the participant to complete and return by free post to the Study Researcher (this will be a member of the QIB Study Research team for Streams 1 & 2 or a trained member of the NSFT Research Nurse team for Stream 3). In order to monitor which stream participants have been recruited from, the footer of the reply sheet states Stream 1 (Annex 8.1), Stream 2 (Annex 8.2) or Stream 3 (Annex 8.3). All participants also have the option of expressing an interest online www.quadram.ac.uk/motionstudy (Annex 7.1). The Study Researcher will then contact the participant by their preferred method with a view to arranging a telephone call as described in section 8.1.

7.1 Stream 1 Participant Identification

It is anticipated that the study will be adopted on to the NIHR portfolio. CRN Eastern (Division 4) will identify research active Primary Care sites across Norfolk to act as Patient Identification Centres (PIC) sites. The GP or Data Manager from each PIC site will search the electronic patient record system to identify potentially suitable participants using the study inclusion/exclusion criteria. A list of potentially suitable participants is then generated who match the search criteria read-codes.

The PIC site will then post the PIL (Annex 2), the Summary PIS (Annex 4) and the Full PIS (Annex 5) directly to the participant. The participant will then send the Stream 1 reply sheet (Annex 8.1) using the free post envelope directly to the research team or complete the expression of interest form online.

In addition, the CRN Eastern team will also supply PIC sites with study fliers (Annex 6.1) and posters (Annex 6.2) for display in PIC site waiting areas.
Stream 1 will potentially identify participants for cohorts 1, 2 & 3.

7.2 Stream 2 Participant Identification

The BCSP has a process whereby any patient who is identified as requiring further bowel investigation (either from a flexible sigmoidoscopy or a positive FOBt) is invited for an initial colonoscopy as part of the BCSP as described in Section 5.2.1.

As part of routine care, the BCSP team post a NHS health questionnaire to their patients who are on the 1 and 3 year CSP waiting list 10 weeks in advance of their colonoscopy appointment and request that this is posted back to BCSP team. Within this envelope will be a Study Introduction Letter (Annex 3) which briefly introduces the MOTION study and explains that the MOTION Study PIL, Summary PIS and Full PIS will be posted to them within a few days. There will also be a free post envelope for the participant to send the Stream 2 reply sheet (Annex 8.2).

The CSP Health Questionnaire and the MOTION Study information will be sent separately in case of confusion with return envelopes. The MOTION study envelope will have a MOTION Study sticker placed on the back of the envelope so that this prompts the participant to place the study reply sheet in to this envelope.

The Study Researcher will inform the CSP team which of their patients have responded to the study and subsequently given written informed consent for the study. This is so that the CSP team can inform the Study Researcher of when the next routine care colonoscopy will be due as part of the routine CSP (the participant will have consented to this sharing of information between the Study Researcher and the CSP). For non-responders to the study, the CSP team will not contact their patients again for study purposes.

At the point of consenting, it will not be known if any participant from stream 2 has any inflammatory bowel condition (Ulcerative Colitis, Crohn's Disease or Diverticulitis). Following the routine care colonoscopy, if a participant is found to have any of these conditions, they will not be withdrawn from the study.

Stream 2 will potentially identify participants for cohorts 1, 2 & 3 (Figure 3).

The current number of patients (as at January 2019) on the waiting list for a scheduled colonoscopy as part of the CSP are as follows: -

<u>01/01/2019 – 31/12/2019</u>
494 patients due for 1 or 3 year surveillance (1 year surveillance patients, only until June 2019).

<u>01/01/2020 – 31/12/2020</u>
531 patients due for 3 year surveillance.

<u>01/01/2021 – 31/12/2021</u>
340 patients due for 3 year surveillance (3 year surveillance patients, only until June 2021).

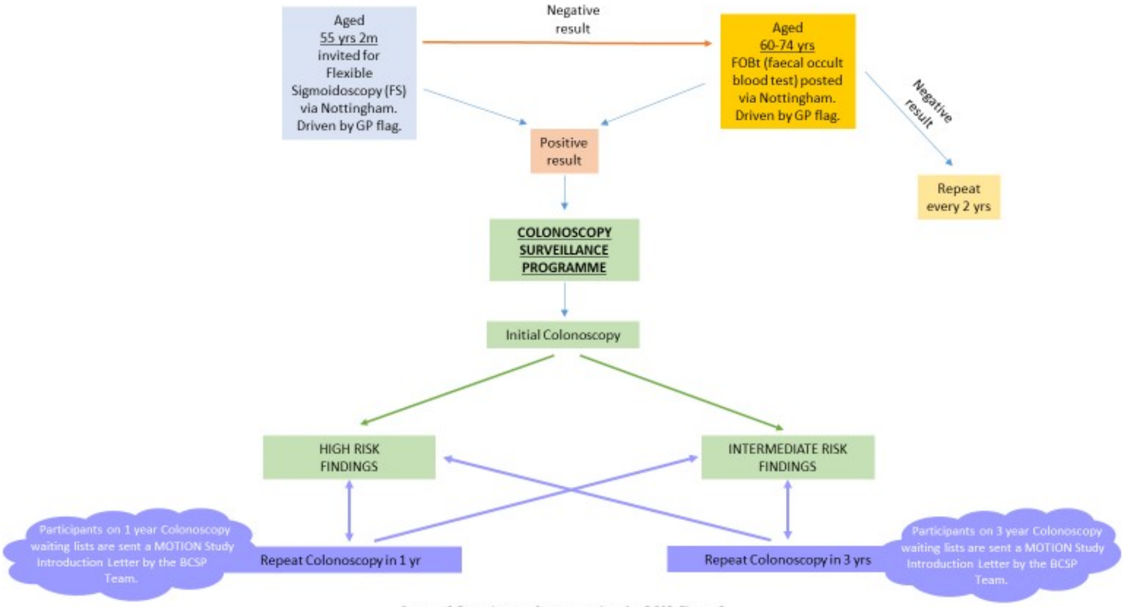

Stream 2 Recruitment Strategy using the BCSP. Figure 3.

7.3 Stream 3 Participant Identification.

Approximately 200 GP referrals per week are received by the Memory Assessment Services in Norwich, South Norwich, North Norwich/Norfolk, Great Yarmouth and Lowestoft. When referred patients are seen for a memory assessment by the Community Teams for each area, they are asked to sign a Consent to Research form (Annex 12). The NSFT Research Team can then approach those participants with a diagnosis of MCI that have completed this form. This team comprises of

experienced research nurses who are led by Claire Rischmiller (Senior Research Nurse).

Through engagement with the NSFT Clinical Teams, MCI participants will be identified in clinics and approached by the Clinical Teams and provided with a brief introduction to the study. They will also be offered the study flier (Annex 6.1). The participant can then request for the study information (PIL, Summary PIS and Full PIS to be sent to them. The participant can then return the Stream 3 reply sheet (Annex 8.3) via the free post envelope directly to the NSFT Research Team.

Collaboration with the NSFT Clinical and Research teams will therefore enable identification of suitable participants who have a diagnosis of MCI and be suitable for cohort 3 and in some instances, potentially cohorts 1 and 2. NSFT links with JDR will also enable identification of suitable participants. All consented participants will undergo the same cognitive testing as part of the screening and final eligibility process in the Pre-Study Visit.
Streams 1 & 2 will not be aware that Stream 3 participants will be seen in their home for the Pre-Study Visit and vice versa.

The overall collaborative recruitment strategy is shown in Figure 4.

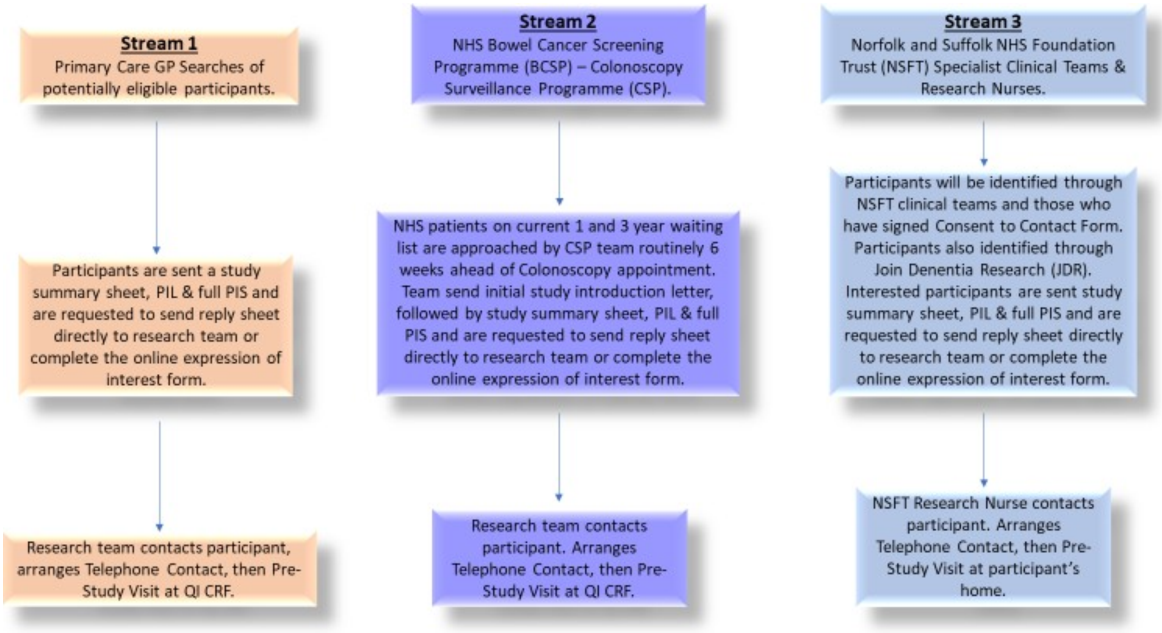

MOTION Study Recruitment Strategy.
Figure 4.

To summarise:

- Streams 1 & 2 will have 10 visits at QI CRF and Stream 3 will have 9 visits at QI CRF (see Section 7.3).
- There are two subgroups in this study (Sections 5.2.1 and 5.2.2).
- All participants will be required to attend Beccles Hospital for OCT/OCT (A) scans (Section 9.1.2) and 30 participants from cohort 3 will undergo brain MRI scans at the NNUH (Section 5.2.2).

7.4 Inclusion Criteria

- Male or female aged at least 60 years.
- Must be able to understand the study and provide signed and dated informed consent.
- Must be able to complete the Cognitive Tests/Questionnaires, Health Questionnaires by themselves and be familiar with using an ipad/tablet. The device will be provided by the Study Researcher and any support required will be available.
- Participants who have MCI would be eligible to participate providing they satisfy all other criteria.
- Over a period of 4 years, participants must be willing and able to attend the QI CRF 10 times. Participants with MCI will be seen in their own home once by a member of the NSFT Research Nurse team for the Pre-Study Visit and will then need to attend the QI CRF 9 times for the remaining study visits.

Participants must be willing and able to do the following at QI CRF: -

- Produce stool samples at home which they will bring to to QI CRF within 24 hours of production.
- Provide blood samples (255 ml in total = 51 teaspoons). There is no requirement to fast for blood sampling.
- Complete health and lifestyle questionnaires (some may be posted from home).
- Undergo height, Tanita Scales Measurement, BP and hand grip strength measurements.
- Undergo cognitive assessment tests.
- All participants will need to attend Beccles Hospital for Optical Coherence Tomography OCT scans. Those participants in subgroup 1 will receive an OCT (A) scan around the Baseline Visit and Study Visit 8.

- 30 participants in subgroup 2 will be requested to undergo a brain MRI at NNUH 4-6 weeks from the Baseline Visit and 4-6 weeks prior to Study Visit 8. This is optional.

It will be explained to all participants that they will be fully supported by the NSFT Research Nurse/Study Research Team for all the procedures, tests and questionnaires that the participant will be required to undertake at each Study Visit. It is acknowledged that the study is complex by nature, therefore where possible, all participants will be encouraged to bring their partner/relative/friend to all the Study Visits to reduce any anxiety.

7.5 Exclusion Criteria

The participant may not enter the study if ANY of the following apply:

- Currently taking part in an interventional study.
- Living with or related to any member of the research team.
- Have a diagnosis of Dementia, Parkinson's Disease, Alzheimer's Disease, Creutzfeldt-Jakob disease (CJD), Picks Disease. **Mild Cognitive Impairment is not an exclusion.**
- Schizophrenia
- Bipolar Disorder
- Obsessive Compulsive Disorder
- Untreated current clinical depression
- Have irreversible brain injury.
- Have had a stroke.
- Have epilepsy.

Any participant who develops any condition listed above in the exclusion criteria following informed consent will be withdrawn from the study. See Section 5.
Any participant who develops any condition listed below in the exclusion criteria following informed consent will not be withdrawn from the study. However, individual cases may be assessed by the study team.

- Take more than a daily dose of probiotics.
- Have a long-standing gastrointestinal or liver function abnormality requiring on-going medical management or medication.
- History of cancer within the last 5 years except for squamous or basal cell carcinomas of the skin that have been medically managed by local excision.

- Unstable dietary history as defined by major changes in diet during the previous month, where a major food group in the diet has been stopped or significantly increased, for example become vegetarian, vegan or stopped eating red meat.
- History of alcohol, drug or substance abuse.
- History of Hepatitis B or Hepatitis C.
- Major surgery of the gastrointestinal tract, apart from gall bladder or appendix removal, in the past five years.
- Any major bowel resection at any time.
- History of Ulcerative Colitis, Crohn's Disease or Diverticulitis.
- Persistent, infectious gastroenteritis, colitis or gastritis, persistent or chronic diarrhoea of unknown cause, *Clostridium difficile* infection (recurrent) or Helicobacter pylori infection (untreated).
- Constipation.
- Regular use of laxatives.

The Full PIS requests that the participant informs the Study Researcher of any change in their medical history. As a secondary measure, the GP Information Letter also requests this information.

8 Phase 1 - Screening and Recruitment Phase

8.1 Telephone Contact

Once a reply sheet from a participant from Stream 1 (Annex 8.1) or Stream 2 (Annex 8.2) is received, they will receive an initial telephone call from the QIB Researcher. If a reply sheet is received from a participant from Stream 3 (Annex 8.3), they will receive the initial telephone call from a NSFT Research Nurse. Each participant will receive an overview of the study requirements and any questions will be answered. The Study Researcher will use the Telephone Contact section of the Telephone Contact/Pre-Study Visit Checklist (Annex 9) as a guide to providing all study information and checking eligibility before making the Pre-Study Visit appointment. It is anticipated that the Telephone Contact call will last approximately 20 minutes. If the participant is unsure of their medical history, they can check this with their GP and let the Study Researcher/NSFT Research team know before the Pre-Study Visit appointment is made.

If the participant has received antibiotics within 14 days, or a vaccination within 28 days of the planned Pre-Study Visit, the Pre-Study Visit will be delayed for the

appropriate washout period (antibiotics is 14 days from the date of the last dose and 28 days from the date of the last vaccination). This will ensure a baseline consistency at the point of consenting at the Pre-Study Visit. Any other circumstances which may delay the Pre-Study Visit from being booked will be discussed on an individual basis.

If the participant is not eligible to participate, they will be given the reason why and thanked for their time and interest in the study.

If the participant is eligible and is happy with the study requirements, the Pre-Study Visit appointment will be made.

For Stream 1 & 2, participants will attend the QI CRF. For Stream 3 participants, they will be seen in their own home by the NSFT Research Nurse as it is acknowledged that they would feel more comfortable. During the Telephone Contact discussion, the participant will be made aware that the Pre-Study Visit will last up to 2.5 hours and that during this time, the Study Researcher/NSFT Research Nurse will do the following: -

- Go through all elements of the study in full detail.
- Go through the Participant Instructions for Stool Collection (Annex 13) and the Participant Stool Sample Collection Log (Annex 14).
- Explain that the purpose of conducting the cognitive tests and associated questionnaires is designed so that memory, reasoning and thinking can be captured over the duration of the study. Show the participant a copy of the Full (Annex 16.1) and Partial Health Questionnaire (Annex 16.2).
- Explain the OCT (A) scans (Stream 2).
- Explain the brain MRI scans with the MRI checklist (Annex 17). The Study Researcher/NSFT Research Nurse will go through this checklist with all participants for consistency.
- Go through the Pre-Study Visit Checklist (Annex 9) to ensure all study information has been provided.
- Show the Data Collection Case Report Form (CRF) (Annex 18).
- Take Informed Consent (Annex 10) for the study and Biorepository Consent (Annex 11) for the long-term storage of samples.
- Explain and deliver the Cognitive test (mini-ACE) and associated questionnaire (CCI) and explain that the tests will form the final eligibility check for inclusion in to the study.
- Provide the Baseline Visit stool collection pack.

- Show and explain the Participant Withdrawal Postcard (Annex 29). If the participant decides to withdraw at any point during the study, they will be asked to complete and send the free post postcard and discard any contents of their collection pack.

The participant will be asked to bring any reading glasses for this appointment and will also be able to have someone accompany them if they wish. For all Study Visits to the QI CRF, mileage at 45p per mile will be paid and car parking charges will be waived. Any taxi or bus fares will be reimbursed upon production of a valid receipt.

8.2 Pre-Study Visit.

It is acknowledged that the Pre-Study Visit is a lengthy process (up to 2.5 hours) so the Study Researcher will schedule in regular comfort breaks that are convenient for the participant. The Study Researcher will follow the structure of this visit as described above.

The Study researcher will go through the consent form with the participant and encourage any questions they may have at this stage; after which the participant will sign the consent form agreeing to participate in the study.

After completing the Informed Consent process (see Sections 11.1), a copy of the completed Study Consent and Biorepository Consent (if applicable) (see section 11.2) will be given to the participant. Copies of all completed consent forms will be posted to the participant if the participant is from Stream 3.

Following completion of the Cognitive Test (mini-ACE) and the associated questionnaire (CCI), the participant will be provided with their Baseline Visit stool collection pack. See Section 11.3.1 for a full description of the Cognitive tests and associated questionnaires. It will be explained to the participant that within 5-10 working days, the anonymised Cognitive Tests will be scored by Professor Michael Hornberger or an experienced Research Assistant within his team, or a member of the Study Research Team to ascertain whether the participant is within the required threshold to be included in the study (see Section 11.3.1 for scoring thresholds).

The participant will then be contacted by the QIB Study Researcher to confirm their final eligibility, by telephone by prior arrangement. If their score does not fall within the required threshold, they will be thanked for their time and interest in the study

(Section 16). If the participant feels worried or anxious because they have any concerns, the Study Researcher will act sensitively and reassure the participant that the tests are for research purposes and are not indicative of any clinical diagnosis. Copies of the completed tests will be routinely sent to their GP (Annex 25) if the participant wishes to discuss them.

If the participant is not eligible to continue because the appropriate cohort for their score already has the required maximum number of 120 participants, they will be withdrawn from the study as per Section 16. Participants will be told that they did not reach the required threshold to be enrolled in the study, as they will not be aware of the cohorts. Arrangements will be made for the Study Researcher to pick up the stool collection pack.

If the participant is eligible to continue, an appointment will be made for their Baseline Visit at QI CRF. The Study Researcher will also discuss the OCT (A) scan appointment and brain MRI (if in cohort 3 and if applicable).

A summary of Phase 1 is shown in Figure 5.

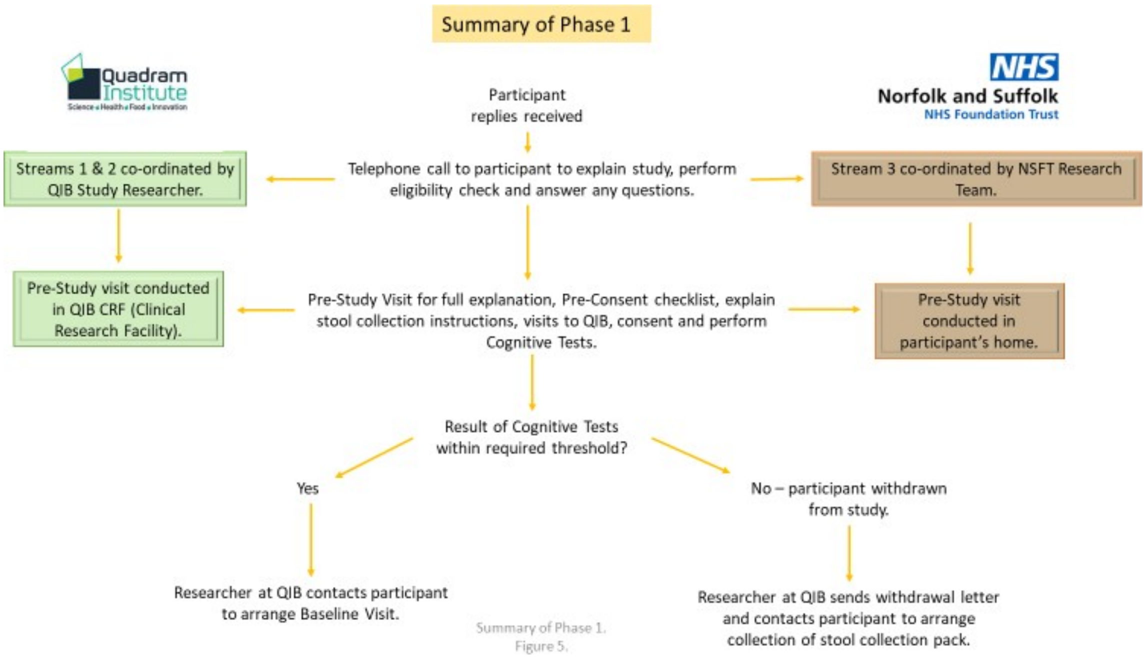

Summary of Phase 1.
Figure 5.

9 Phase 2 - Sample and Data Collection Phase

Participants will be sent a standard reminder (by their preferred method) about their next Study Visit within one week in advance of their appointment (Annex 33).

They will also be reminded to bring a list of all their current prescribed and over-the-counter medication. The participant will be asked to let the Study Researcher know if they are taking or have taken antibiotics within one week of their scheduled appointment. In this case, the appointment will need to be rescheduled as a two-week washout is required from when the last dose of antibiotics was taken. In addition, they will also be reminded to let us know of any travel or flu vaccinations scheduled within 28 days of their appointment as a 28-day washout is required. The participant can record these on their Study Appointment Schedule (Annex 19). At the start of each study visit, the participant will be asked if they are still happy to continue with the study.

All study-related travel and car parking costs to QI CRF, NNUH and Beccles Hospital will be reimbursed to the participant at the end of each study visit. Mileage will be reimbursed at the current QIB standard rate of 45p/mile. Taxi and bus fares will be reimbursed on production of a valid receipt. Light refreshments and comfort breaks will be offered.

9.1 Study Visits

9.1.1 Baseline Visit (approximately up to 3 weeks from Pre-Study Visit).

The participant will be provided with their Study Appointment Schedule (Annex 19) and a Study Identification carry card (Annex 20). The Study Researcher will go through these with the participant. The participant will also receive a freezer magnet displaying the study logo. This will act as a reminder for the participant to freeze their ice-packs ahead of stool sample collection. Participants will be informed that they will be sent a one-page newsletter either via an online link (Annex 38) or provided as a hard copy at the appropriate study visit to provide recruitment updates, study reminders and sign post participants to related information of interest.

This visit will take approximately 2 hours. Participants will be required to attend the QI CRF for their Baseline Visit for the following: -

- Undergo Cognitive tests and associated Questionnaires (see Section 11.3.1 for full description): -

  - Mini ACE – repeated again at this visit for reliability
  - CCI – repeated again at this visit for reliability

- PHQ 9 (Patient Health Questionnaire)
- Supermarket Test
- Sea Hero Test
- CBI-R (participant)
- RCFT (Rey Complex Figure Test)
- TMT (Trail Making Test)
- BPQ (Body Perception Questionnaire)

The CCI, PHQ9, CBI-R and BPQ are self-administered tests. The remainder activities are delivered by the Study Researcher.

- Complete the Full Health Questionnaire.
- Obtain Blood sample – 40 ml (8 teaspoons).
- Collect physical measurements (height, Tanita scales measurement, BP and hand grip strength measurements).
- Discuss the OCT/OCT (A) scan and brain MRI scan (as appropriate) appointment as below. Participants will be made aware that they would need to allow up to 1.5hrs for the brain MRI and up to 3 hours for the OCT/OCT (A) scan.

9.1.1.1 Baseline brain MRI scan appointment

The Baseline Visit brain MRI scan appointment will be scheduled within 6 weeks of the Baseline Visit, and the appointment letter (Annex 24) will be sent by the Study Researcher along with the NHS MRI Safety Questionnaire (Annex 17). The participant will also receive a reminder (by their preferred method) from the Study Researcher within one week of when the appointment is due (Annex 35).

The MRI appointment/follow-up process flow is shown in Figure 6.

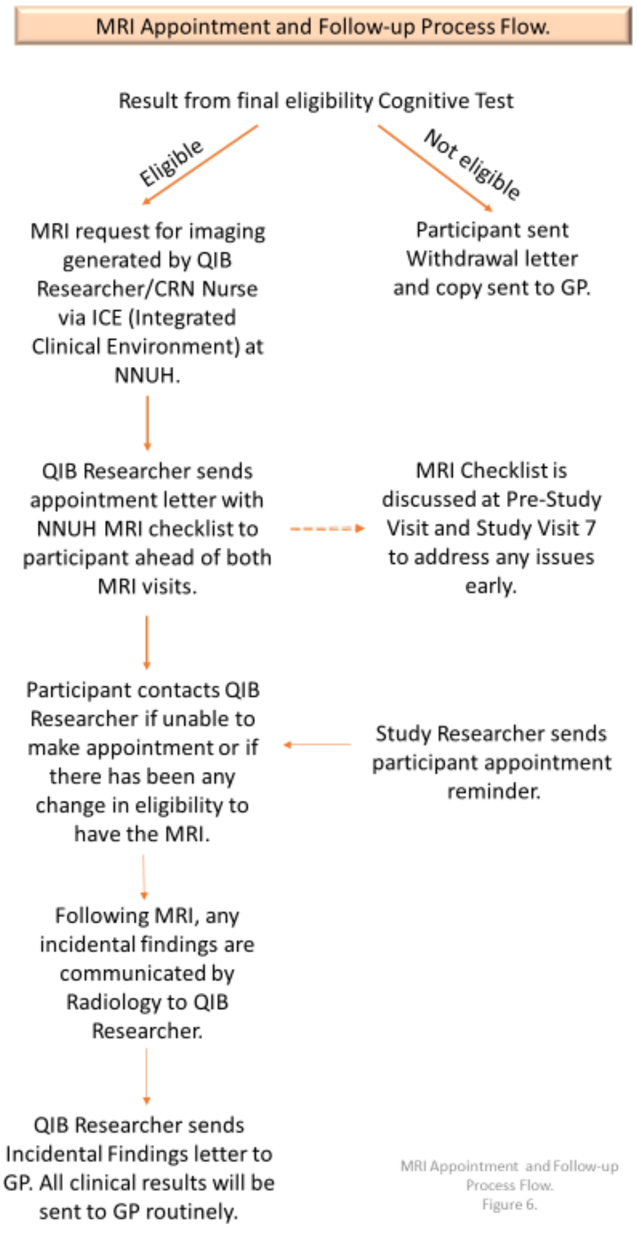

MRI Appointment and Follow-up Process Flow.
Figure 6.

9.1.1.2 Baseline OCT/OCT (A) scan appointment

The Baseline OCT/OCT (A) Scan appointment at Beccles Hospital will be scheduled for within one month of the Baseline Visit. The appointment letter (Annex 22) will be sent from Beccles Hospital and will routinely request participants to bring in a copy of their current prescriptions. This letter will be sent with an OCT/OCT (A) Patient Information Sheet (Annex 23) generated by Beccles Hospital.

The participant will also receive a reminder (by their preferred method) from the Study Researcher within one week of when the appointment is due (Annex 34).

The OCT/OCT (A) appointment and follow-up process flow is shown in Figure 7.

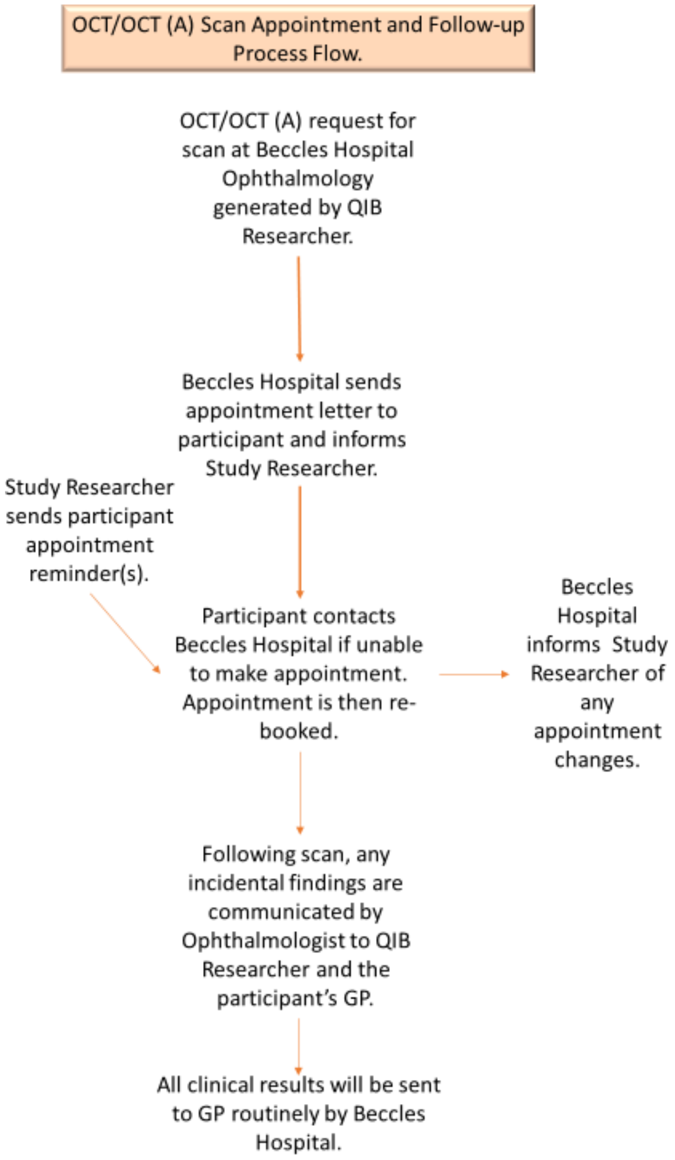

OCT/OCT (A) Scan Appointment and Follow-up Process Flow.
Figure 7.

9.1.2 Study Visits 1, 3, 5 & 7

Study Visit 1 will be 6 months from the Baseline Visit.
Study Visit 3 will be 18 months from the Baseline Visit.
Study Visit 5 will be 30 months from the Baseline Visit.
Study Visit 7 will be 42 months from the Baseline Visit.

These visits will take approximately 2 hours. Participants will be required to attend the QI CRF for the following at each visit: -

- Drop off the cool box containing their stool sample.
- Pick up any replacement stool collection items ahead of next Study Visit.
- Blood sample – 15 ml (3 teaspoons).
- Complete the Partial Health Questionnaire.
- Undergo Cognitive tests and associated questionnaires: -
- Mini ACE
- CCI
- PHQ 9 (Patient Health Questionnaire)
- Supermarket Test
- Sea Hero Test
- CBI-R (participant)
- RCFT (Rey Complex Figure Test)
- TMT (Trail Making Test)
- BPQ (Body Perception Questionnaire)

The Study Researcher will explain the following at the end of each of the following Study Visits:-

- Study Visit 3 - remind the participant that they will receive an appointment letter from Beccles Hospital for their OCT scan, which will be scheduled to take place within one month before or one month after Study Visit 4.
- Study Visit 7 - remind participant that they will receive an appointment letter from Beccles Hospital for their OCT (A) scan. This appointment will be scheduled for around 4 weeks before Study visit 8 and will take place at Beccles Hospital. If applicable, the Study Researcher will go through the MRI checklist and remind participant that they will receive an appointment letter for a MRI scan at NNUH around 6 weeks before Study Visit 8. Both scans will be scheduled prior to Study Visit 8 where possible so that the participant can be reimbursed for any travel and parking costs at Study Visit 8 (final visit).

9.1.3 Study Visits 2, 4, 6 & 8

Study Visit 2 will be 12 months from the Baseline Visit.
Study Visit 4 will be 24 months from the Baseline Visit.
Study Visit 6 will be 36 months from the Baseline Visit.
Study Visit 8 will be 48 months from the Baseline Visit.

These visits will take approximately 45 mins. Participants will be required to attend QI CRF for the following at each visit: -

- Drop off the cool box containing their stool sample.
- Pick up any replacement stool collection items ahead of next Study Visit (apart from Study Visit 8).
- Blood sample – 40 ml (8 teaspoons).
- Physical measurement collection (height, Tanita Scales Measurement, BP and hand grip strength measurements).
- Complete the Partial Health Questionnaire.

The Study Researcher will provide the following at the end of each of the following Study Visits:-

- Study Visit 4 - as a thank you for their participation so far, each participant will receive a Love2shop voucher to the value of £10.
- Study Visit 8 - the participant will be thanked for their participation in the study and given a further Love2shop voucher to the value of £15 for their time and any inconvenience.

9.1.4 Time windows for Study Visits

At each Study Visit, participants will be provided with their next scheduled appointment at QI CRF but can be seen up to one month before or one month after this date.

At the end of the study, eFI (electronic Frailty Index) scores will be requested retrospectively from the participant's GP (Annex 31). See Section 15.3.

Figure 6 below shows a summary of Phase 2.

Participation	PHASE 2								
	Baseline	Study Visit 1	Study Visit 2	Study Visit 3	Study Visit 4	Study Visit 5	Study Visit 6	Study Visit 7	Study Visit 8
	0 months	6 months	1 year	1.5 years	2 years	2.5 years	3 years	3.5 years	4 years
		1 month either side	1 month either side	1 month either side	1 month either side	1 month either side	1 month either side	1 month either side	1 month either side
Drop off stool sample at QIB	X	X	X	X	X	X	X	X	X
Pick up stool collection pack for next visit	X	X	X	X	X	X	X	X	
Cognitive Tests and related Questionnaires	X	X		X		X		X	
Full Health Questionnaire	X								
Partial Health Questionnaire		X	X	X	X	X	X	X	X
Blood Sample	X	X	X	X	X	X	X	X	X
Physical Measurement Collection	X		X		X		X		X
Optical Coherence Tomography (Retinal) Scan	X				X				X
Brain MRI (optional)	X								X
Electronic Frailty Index Data from GP									X
Colonoscopy Tissue Biopsy	IF INVITED FOR A COLONOSCOPY AS PART OF THE NHS BOWEL CANCER SCREENING PROGRAMME								

Summary of Phase 2

Figure 6

10 Incidental Findings

During review of all results sent to the GP as detailed below, any clinically relevant incidental findings will be discussed with the participant by their GP.

10.1 Cognitive Tests

All cognitive tests and associated questionnaires will be sent to the participant’s GP. Participants will be aware that the mini-ACE cognitive test is used as a tool in the clinical setting to diagnose dementia, but that it will only be used in combination with all other cognitive tests/associated questionnaires for research purposes. Copies of the mini-ACE and CCI test from the Pre-Study Visit will be sent to the participant’s GP. The GP will also receive copies of cognitive tests and associated questionnaires taken at all the specified time points.

10.2 MRI Scans

Dr Janak Saada or a member of the Radiology team at NNUH will inform the Clinical Studies Officer at QIB of any incidental findings following the participant's MRI scan. The NNUH team will follow hospital standard procedures for incidental findings. The participant's GP will be sent a letter when a brain MRI scan has been performed (if applicable) along with a copy of the result.

10.3 OCT/OCT (A) Scans

Dr Ben Burton or a member of the Ophthalmology team will send a summary of the scan results which will include any incidental findings directly to the participant's GP. An anonymised copy will also be sent to the Clinical Studies Officer at QIB.

10.4 Full Blood Count, Biochemistry and Troponin C blood results

The Full Blood Count (FBC), Biochemistry and Troponin C samples will be analysed at the NNUH accredited Department Laboratory Medicine. The participant's GP will be sent a letter along with copies of all blood results at the appropriate time point.

10.5 Colonoscopy Biopsies

Any findings following routine care biopsies will follow the NNUH Trust policy.

11 STUDY PROCEDURES

The Schedule of Events is provided as Appendix A in Section 27.

11.1 Informed Consent

The PIS and Informed Consent Form will be presented to the participant at the Pre-study Visit detailing no less than the exact nature of the study; what it will involve for the participant; the implications and constraints of the protocol; the known side effects and any risks involved in taking part. It will be clearly stated that the participant is free to withdraw from the study at any time for any reason without prejudice to future care, without affecting their legal rights, and with no obligation to give the reason for withdrawal.

The participant will be allowed as much time as wished to consider the information, and the opportunity to question the Study Researcher, their GP or other independent parties to decide whether they will participate in the study. Written

informed consent will then be obtained using the Study Consent Form (Annex 10) by means of participant dated signature and dated signature of the Study Researcher. The Study Researcher obtaining informed consent will be GCP trained be suitably qualified and experienced, have been authorised to do so by the Chief Investigator and will appear in the Delegation of Duties Log. A copy of the signed Informed Consent will be given/sent to the participant. The original signed form will be retained at the Sponsor site. A copy will be sent with the Summary PIS and the Full PIS to the participant's GP (Annex 21) and for insertion in to the participant's hospital notes along with the appropriate Full PIS. The Consent form will ask for explicit consent to have access to their GP and hospital notes for study-related activities.

Consent is an ongoing process and as such will be confirmed at each contact or visit by verbal means.

11.2 Informed Consent for Biorepository Storage

Following on from Informed Consent for study participation, the participant will be asked if they would be happy for the samples taken to be stored (anonymised) at -80° in the Norwich Biorepository during the study and after the end of the study. The Norwich Biorepository is a NNUH tissue bank where research samples taken as part of the study can be used by other researchers externally for future ethically-approved research. No additional samples will be taken for this purpose. The participant will be asked to read the current version of the NNUH Adult Tissue Bank Information Sheet and to sign this consent if they wish. This will be detailed in the PIS.

11.3 Study Specific Tests

11.3.1 Cognitive Tests

Cognitive tests are designed to measure memory, reasoning and thinking. This study is looking at the analyses of data and samples of participants who are deemed to be at low (no subjective and no objective cognitive deficit), medium (subjective but no objective cognitive deficit) or high (subjective and objective cognitive deficit) risk of developing dementia. Subtle objective cognitive deficits indicative of the prodromal stages of dementia are referred to as mild cognitive impairment (MCI) [26]. The MCI identifies currently healthy ageing individuals at high risk of

developing dementia. MCI does not mean a diagnosis of dementia as up to 40% of MCI patients revert back to healthy after 12 months.

In terms of diagnosing dementia in the clinical setting, there is no standardisation across the NHS. Most of the cognitive tests and associated questionnaires that will be used in this study are used in the clinical setting for diagnosing dementia, mainly the mini-ACE test, but there is variation in the use of these across dementia services nationally. The Full PIS states that the mini-ACE test is used in the clinical setting as a tool for diagnosing dementia, but for the purposes of this study, the mini-ACE, RCFT, CBI-R, PHQ 9 and TMT are used for recording different levels of cognition over time and are used in combination with each other only for the purposes of this research study and not for diagnostic purposes. See Section 15.1 for explanations of why individual cognitive tests and associated questionnaires are being used in this study.

Participants will be required to undergo a cognitive test (mini-ACE) and an associated questionnaire (CCI) following Informed Consent at the Pre-Study Visit. Final eligibility will be determined by Professor Hornberger or his experienced Study Researcher/Research Assistant using pre-determined thresholds for each cohort as detailed below.

❖ Cohort 1 (low risk) - No subjective and no objective deficit = mini-ACE score of ≥26/30 and CCI score of <16 from the first 12 questions.

❖ Cohort 2 (medium risk) - Subjective and no objective deficit = mini-ACE score of ≥26/30 and CCI score of ≥16 from the first 12 questions.

❖ Cohort 3 (high risk) - Subjective and Objective deficit = mini-ACE score of <26/30 and CCI score of ≥16 from the first 12 questions.

For the purposes of this study, the high risk group will be referred to as having MCI.

Participants will then be assigned to one of the three cohorts based on these scoring thresholds. Results of the cognitive tests will not be discussed with participants as we do not wish to cause any undue anxiety or concern. Copies of completed cognitive tests and associated questionnaires will be sent to the participant's GP. Participants who do not fall within the threshold for that cohort, or if the appropriate cohort already has the maximum number of 120 participants, they will

be withdrawn from the study (see section 16). For any participant who is found to have an incidental finding of MCI, please see Section 10.1.

The NSFT Research Nurse team and the Study Researchers delivering the Cognitive Tests and associated questionnaires will have received full training from experienced Research Assistants.

1. Mini Addenbrooke's Cognitive Examination (Mini-ACE) [27] is a short 5 min standard cognitive screening test well validated and used in dementia (Annex 15.1).

2. Cognitive Change Index (CCI) [28], a validated self-completed measure of perception of the participant's cognitive decline (Annex 15.2). This is will take approximately 5 mins.

In addition to the above, the following 7 Cognitive tests will also be performed at Study Visits 5, 7, 9 & 11:

3. Patient Health Questionnaire 9 (PHQ-9) [29], a validated, brief self-report multipurpose instrument for screening the severity of depression symptoms in the participant (Annex 15.3). This will take approximately 5 minutes.

4. Supermarket Task [30], a non-validated computer- and tablet-based assessment of spatial orientation using an ecological supermarket environment (Annex 15.4). This will take approximately 15 minutes.

5. Sea Hero Quest (SHQ), a non-validated novel computer, tablet, or virtual reality measure of spatial navigation within the context of a video game and takes 15 minutes to complete (Annex 15.5).

6. Cambridge Behavioural Inventory Revised (CBI-R) [31] is a validated self-rated measure of changes in the participant's behaviour. These domains include memory and orientation, everyday skills, self-care, abnormal behaviour, mood, beliefs, eating habits, sleep, stereotypic and motor behaviours and motivation and frequency of these behaviours (Annex 15.6). This takes around 15 minutes to complete.

7. The short version of the Body Perception Questionnaire [32], a non-validated, self-report questionnaire assessing the participant's degree of interoception ability (Annex 15.7).

8. The Rey Complex Figure Test (RCFT) [33], a validated short (10 min) measure of visual memory and visuospatial constructional ability which includes a 3-minute delayed recall (Annex 15.8).

9. The Trail Making Test (TMT) [34], a validated short (5 minute) test of processing speed, attention and set-switching (Annex 15.9).

11.3.2 Optical Coherence Tomography (OCT) Scans

There is a growing literature on the use of OCT scanning to measure both the Retinal Nerve Fibre Layer (RNFL) and the Inner Ganglion Cell Layer (iGCL) of the retina and correlating these measurements with the risk of dementia [17, 35]. Embryologically the eye is an out pouching from the brain and contains neural tissue. It is possible that if the brain is undergoing a degenerative atrophy as seen in dementia then there might also be measurable changes in the retina, particularly the retinal nerve fibres of the ganglion cells. This might mean that OCT of the RNFL or iGCL becomes a useful non-invasive way of detecting those at higher risk of dementia, or quantitatively measuring the response to novel treatment strategies. The OCT scan will be performed following the Baseline Visit, around Study Visit 4 and prior to Study Visit 8 in all participants. Consistent with this speculation is the very recent finding microvascular changes in the retina (foveal thinning) detected by using OCT angiography align well with imaging and biomarker evidence of preclinical Alzheimer's disease [36]. The OCT and retinal colour photo data collected will also detect age related macular degeneration and may allow us to correlate the presence or absence of AMD to the gut microbiome [37].

11.3.3 Optical Coherence Tomography Angiography OCT (A) Scans

We are also going to use the much newer technique of OCT (A) at the Baseline Visit & Visit 8 in subgroup 1 (the same 90 participants – 30 from each cohort who undergo a colonoscopy) which, in addition to the OCT scan, measures blood flow in the retina, again in a non-invasive manner. We wish to get OCT (A) scans on the participants in whom we have the most information about the gut microbiome and

this will be those in Subgroup 1. Ideally, we would wish to have OCT (A) in all participants but funding limitations mean this is not possible. In order to make meaningful conclusions from the OCT (A) data we need to compare participants from each cohort so arranging OCT (A) on the 90 participants in subgroup 1 seems a sensible compromise.

There is minimal data on correlating OCT (A) findings with dementia, or indeed how to quantify and analyse the large amount of data that OCT (A) scans generate [38].

However, the MOTION study is unique in providing an opportunity to collect OCT (A) data in ageing participants with and without MCI which will be examined by a masked observer to see if there is any change in the vascular pattern in participants at risk of developing dementia (cohort 3), looking at both the retinal and choroidal blood supply, measuring when possible the vascular density and looking for other changes such as enlargement of the foveal avascular zone which might reflect progressive microvascular changes.

11.3.3.1 OCT (A) Scan Protocol

All participants recruited into the study will be offered a retinal OCT scan, colour photography and ophthalmic examination.
The following information will be collected: -

11.3.3.2 History

- Patient ID/Study number
- Age
- Ophthalmic history
- Ophthalmic medication including vitamins to prevent age related macular degeneration
- Any allergies

11.3.3.3 Examination

- Best corrected Visual acuity by ETDRS chart in each eye
- Ishihara colour vision in each eye
- Slit lamp examination
- Intra-ocular pressure by Goldman Tonometry
- Right eye (or left eye if right eye not suitable) will be dilated using tropicamide 1% eyedrop.

- Clinical examination of retina and optic nerve to pick up any confounding pathologies such as retinal vein occlusion, age related macular degeneration and glaucoma.

If participants decline dilating drops the examination will continue and we will document that they were not dilated in either eye.

11.3.3.4 Investigations at every Ophthalmology visit

For all participants (n=360), acquisition protocol using SDOCT Spectralis OCT2 system will include the following methodology at the Baseline Visit, Study Visit 4 and Study Visit 8.
Retinal nerve fibre layer (RNFL) scans will be acquired with the standard peripapillary (pRNFL) scan pattern, at 100 frames Automatic Real-Time Tracking (ART) setting, by an OCT technician employing FOBMO (fovea to Bruch's membrane opening) axis orientation.
For all study participants, Posterior pole scans on glaucoma setting, will be used to obtain a 30 by 25 degree macular volume scan pattern, using 16 ART frames. This is a horizontal 61-line volume scan available as a standard OCT scan pattern on OCT2 system (with an interscan spacing interval of 120 microns). Built-in Spectralis (Heyex software version 6.9) will be used for automated retinal layer segmentation to report on the total retinal thickness, and individualized retinal layer thicknesses for ETDRS rings centred on the fovea and compared with available normative database. The individualized retinal layer thicknesses will be reported for RNFL (retinal nerve fibre layer), GCL (Ganglion cell layer), IPL (Inner Plexiform Layer), INL (Inner Nuclear Layer) OPL (Outer Plexiform Layer), OPL (Outer Plexiform Layer) and RPE (Retinal Pigment Epithelium Layer), as available with proprietary Heyex software (version 6.9 Heidelberg Engineering Ltd, Germany).

At all eye clinic study visits, Choroidal thickness will be measured using EDI (Enhanced depth imaging) cross-hair imaging at 100 ART frames setting. SFCT (sub-foveal choroidal thickness using the manual caliper between the two measurement points with the inner border at RPE and outer border at CSI (Chorio-scleral interface). Colour retinal photography of optic disc and Macula will be performed.

11.3.3.5 OCT Angiography - OCT (A) scans

OCT (A) using Spectralis OCT2 system uses a centre wavelength of 870 nanometers with an A-scan frequency of 85,000 scans/s to obtain a volume at 6 microns spacing with a macular field of view of 3 * 3 mm. Proprietary real time True track technology

is used by Heidelberg system, with 2-5 times B-scan repetition at the same retinal location to generate OCT (A) signal employing full-spectrum amplitude decorrelation software developed by the Heidelberg engineers. OCT (A) analysis will report on FAZ area (foveal avascular zone) using the built-in area measuring caliper, Vascular density indices of SCP (Superficial Capillary Plexus), ICP (Intermediate Capillary Plexus) and DCP (Deep Capillary Plexus).

Any scans of poor quality due to any reason, for example media opacity due to cataracts and less than Q (quality factor) of 20 will not be used for analysis.

11.3.3.6 Main Outcome Measures

Peripapillary RNFL in superior, inferior, temporal and nasal quadrants and mean quadrants. Macular thickness will be recorded form the OCT and inner Ganglion cell layer thickness will be recorded by outer, inner and foveal segments.

Ishihara test score. Choroidal thickness will be recorded from the OCT. OCT (A) data will be analysed for area of Foveal Avascular Zone (FAZ).

Data reported from the right eye unless the OCT grader reports segmentation failure. In that case, data from the other eye will be used if no segmentation failure is recorded for that eye.

Participants with ophthalmic conditions which clearly interfere with the OCT measurements will be excluded from the analysis. This would include diabetic retinopathy, retinal vein occlusion, uveitis and glaucoma.

11.4 Brain Magnetic Resonance Imaging (MRI) Scans.

If the participant is in the sub-group for MRI brain imaging, these will be performed at the NNUH by Dr Janak Saada (Radiology Consultant) or a member of his team. The analysis of this data will be performed by Professor Hornberger following standard protocols.

Scans will be performed to allow for the cognitive and behavioural data collected at the previous visits to be related to neuroanatomical changes. The 60-minute scan will capture both structural and functional sequences.

Information regarding the safety and comfort relating to MRI scanning will be provided in the PIS, and participants will have the option to consent to this component of the study in the Informed Consent Form. If participants elect to undergo the MRI scan, they will be screened prior to scanning for any metal in the

body or other contraindications to scanning, including claustrophobia (this element is included in the Pre-Study Visit Checklist).

The scanning procedure will involve participants having their head scanned within the MRI machine. They will be provided with earplugs and additional ear protection from the noise of the scanner, as well as pillows, foam padding, and blankets to ensure comfort during the scan. They will be provided with a button to alert MRI staff of any complications or discomfort during the scan, and the scan will be aborted immediately at any time if the participant wishes. There are no known adverse side effects with having a single or multiple MRI scans of any length, even repeated scans after short intervals provided that the participant does not have any metal within or on the body. The MRI scans do not involve injection of radioactive compounds or exposure to potentially harmful radiation.

It is possible that during testing unexpected abnormalities may be found that may require further medical attention. For example, an 'incidental' finding of a brain tumour on MRI scanning or any other risks beyond the scope of the study. This is unlikely as the participants are considered healthy at the time of the study. However, in the event of this happening the participant will be informed and with their consent, their GP who will be asked to take further action as they see necessary. Participants will also consent to having their GP informed of any relevant information, including information raising concerns about their wellbeing, before they commence participation in the study. Participants can opt out for personal or medical reasons and this will not affect their ability to continue in the study. Any findings following MRI scanning will follow the NNUH Trust policy.

11.4.1 MRI Analysis

The 3D T1-weighted sequences will be acquired as follows: coronal orientation, matrix 256 _ 256, 200 slices, 1 x 1 mm in-plane resolution, slice thickness 1 mm, TE/TR = 2.6/5.8 msec. 3D T1-weighted sequences will be analysed using FSL and SPM software packages. Following brain extraction, tissue segmentation will be carried out using FMRIB's Automatic Segmentation Tool (FAST). The resulting grey matter partial volume maps will be aligned to the Montreal Neurological Institute standard space (MNI52) using the nonlinear registration approach with FNIRT which uses a b-spline representation of the registration warp field. To correct for local expansion or contraction, the registered partial volume maps will be modulated by dividing them by the Jacobian of the warp field. The Jacobian modulation step will

not include the affine part of the registration, which means that the data will be normalized for head size as a scaling effect. The modulated images will be then smoothed with an isotropic Gaussian kernel with a standard deviation of 3 mm (FWHM: 8 mm). This will be followed by GLM and Bayesian statistics on a regional and whole brain level.

11.5 Blood sampling

A trained member of the Research Team will take the following samples at the QI CRF for which the participant will not be required to fast:-

- 2 x 9.5ml (8 teaspoons) of blood will be taken for serum using serum separation tubes.
- 2 x 10ml for plasma using EDTA tubes.

These will be taken at the Baseline Visit and Study Visits 2, 4, 6 & 8. Samples will be analysed at QIB.

- 4ml of blood will be taken for a Full Blood Count (FBC) panel.
- 5ml sample taken for Biochemistry panel.
- 6ml sample taken for Troponin C.

These will be taken at Study Visits 1, 3, 5 and 7 and samples will be analysed at the NNUH.

<u>Participants will provide 255ml of blood (51 teaspoons) in total over 4 years for the purpose of this study</u>.

11.6 Health Questionnaires.

A Full Health Questionnaire (14 pages) requests information on the participant's diet and lifestyle. Participants will be asked to complete this questionnaire at their Baseline Visit at the QI CRF. Participants will be then requested to complete a Partial Health Questionnaire comprising of 5 pages at each of their follow-up visits (Study Visits 1 – 8) at the QI CRF.

11.7 Physical Measurement Collection (Height, Tanita Scales Measurement, BP (blood pressure) and Hand Grip Strength Measurements).

These measurements will be taken at the QIB CRF by a trained member of the Research Team at Study Visits 2, 4, 6 & 8. Height, BPs (taken once in each arm) and

hand grip strength measurements will be recorded manually on the Data Collection Case Report Form by the Study Researcher. The Tanita scale's body composition measurements will be taken with the participant's shoes and socks removed and by standing on the Tanita scales. The scales then provide an electronic print-out which the Study Researcher will add the participant's study identification number to. The print-out will then be photocopied and attached by stapler to the back of the DC CRF. The original print-out will then be stapled to the photocopied version.

11.8 Drop-off & collection of the stool collection pack.

The Baseline Visit stool collection pack will be given to the participant at the end of the Pre-Study Visit and the Study Researcher providing this will go through the collection instructions with the participant.

11.8.1 Contents of stool collection pack

- Cool box.
- 3 x ice-packs measuring approx. 12cm by 13cm by 3cm.
- FaecesCatcher (device for collecting stool sample).
- Collection bags and gloves.
- Anaerogen sachet.
- Outer biohazard bag.
- Participant Sample Collection Log.

At the start of each of the follow-up visit appointments at the QI CRF (Baseline Visit - Study Visit 8), the Study Researcher will meet the participant and the cool box containing the stool sample and will be given to the experienced study Laboratory Technician for processing. The participant will be provided with their next collection pack containing all the stool collection equipment at the end of each visit appointment (apart from Study Visit 8). The Study Researcher will ensure that the participant understands the collection instructions at each visit.

12 SCREENING AND ELIGIBILITY ASSESSMENT

- Stream 1 participants from Primary Care will be sent the appropriate study pack by their GP who will have used the inclusion/exclusion search criteria to search for suitable participants.

- Stream 2 participants will be from the NNUH CSP.
- Stream 3 participants will be from the NSFT.

All participants will receive an initial Telephone Contact call by the Study Researcher. As part of this conversation, the Study Researcher will go through the PIS which lists all the inclusion/exclusion criteria.

At the Pre-Study Visit, all participants will then undergo the following Cognitive Test and associated questionnaire to assess final eligibility: -

1. Mini Addenbrooke's Cognitive Examination (Mini-ACE).
2. Cognitive Change Index (CCI).

13 SAMPLE HANDLING

13.1 Collection, Processing, Laboratory Analysis & Storage

Sample collection is fully described in Section 27. Only QIB-based researchers that are members of the Gut Microbes and Health strategic research programme that are directly involved with this study will access samples.
All samples will be anonymised and stored at -80°C until the end of the study. Any unused samples will be stored in the Norwich Biorepository and used in ethically approved studies in the future.

13.1.1 Colon tissue Biopsy

Why are we collecting these samples?
Microbe populations that are specifically and intimately associated with the lining of the gut wall are ideally situated to influence the host and intestinal cells, but they can be underrepresented or absent in stool samples. For this reason, colon tissue samples are important to: 1) fully identify the microbes that are in most intimate contact with the host 2) identify and analyse how the cells within the tissue respond and react to these microbes and their products.

For colonoscopy biopsies, 6-8 pinch biopsies each measuring 3mm^3 will be taken from the large bowel during the CSP colonoscopy. These biopsies are taken to examine the microbiome that is adhering directly to the mucosal lining of the colon.

13.1.1.1 Colon Tissue Biopsy Laboratory analyses

Tissue will be used immediately and/or preserved in long-term storage in the Norwich Biorepository. Fresh tissue will be placed directly into a plastic tube or in sterile media (e.g. RPMI, HBSS or media for *in vitro* organ culture [IVOC]) for immediate use in organ or cell culture systems, or for cell isolation and analysis. For organ culture, QIB has recently developed and optimised protocols for polarised IVOC and for isolating epithelial stem cell containing crypts from the intestinal epithelium dissected from biopsy samples. When placed into culture in media containing specific growth factors these stem cell-containing crypts faithfully generate intestinal epithelial organoids containing all of the different cell types found *in vivo*. This organ culture system provides a unique, powerful and accessible *in vitro* system to investigate microbe-host (epithelial) cell interactions in the gut. In addition, QIB is also developing microfluidics-based organ-on-a-chip technology with the aim of incorporating intact biopsy samples or cells derived from them (e.g. organoid cultures) into the chips. By linking in sequence gut chips with others containing brain-derived cells we will be able to investigate experimentally the pathways and mediators of gut-brain connectivity and the role gut microbes play in this process. This will be facilitated by incorporating in line monitoring (e.g. electrophysiology measurements) and sampling (of input and output channels) into the chip design. Other immediate means of processing tissue include those for (immuno) histology; isolating cells (e.g. immune and epithelial cells) for detailed characterisation (e.g. repertoire and subset identification and functional properties); proteins isolation/quantification (e.g. cytokines and other inflammatory or immunoregulatory proteins); and/or nucleic acids (DNA/RNA) extraction.

We will undertake targeted gene expression analysis from tissue biopsies. Total RNA will be extracted from fresh and frozen samples in commercially available RNALater solutions. RNAlater product ‘freezes’ gene transcription and stabilises the RNA present so that it can be extracted at a later stage. Total RNA isolation will be performed using RNeasy kits (Qiagen) according the manufacturer’s instructions following RNA later treatment; these are used routinely for the extraction and purification of RNA. Alternatively, RNA and proteins will be extracted from biopsy pre-treated with TRIzol® Reagent that allows the isolation of RNA, DNA, and protein from the same sample.

RNA extracted from the biopsy samples will be concentrated and the quality assessed using Agilent technologies lab-on-a-chip RNA 6000 nano assay. A selection

of genes, including but not limited to mucin, glycosyltransferases, tight junction proteins, serotonin transporter, tryptase, and protease-activated receptor 2 will be analysed by Real-Time RT-PCR. The methods are established in our laboratory at QIB and specific SOPs (Standard Operating Procedures) will be followed for the purpose of this study.
DNA will be extracted from tissue samples using the QIAamp DNA Mini Kit (Qiagen) according to the QIAamp DNA Mini Kit tissue protocol. DNA will be used to assess the composition of mucosa-associated microbiota and to determine selected genotypes considered relevant for the purposes of this study.

Genetic analyses performed during the study will not have any clinical relevance to participants or their relatives, therefore, participants will not receive any results of these tests.

For long term storage, biopsy samples will be preserved by, for example, snap freezing in liquid nitrogen (particularly useful for preserving RNA) and/or immersion in formalin (ideal for preserving tissue histology).

13.1.2 Stool sample

Why are we collecting these samples?

Gut microbes influence many important processes in the body including the digestion of food, programming of the immune system, and helping to prevent invasion of pathogenic microbes. Importantly, these microbes change during [39]ageing with these changes being associated with declining physical and mental health. Identifying microbes from stool samples allows us to determine: 1) which constituent members of the gut microbiome are changing and when: 2) what relationships there are between these changes and a deteriorating function of other organ systems (e.g. the brain, eyes, heart, liver, kidneys, bone and thyroid): 3) how different lifestyle and behavioural factors (e.g. medications) may contribute to changes in microbe populations.
Stool samples are taken to examine the microbiome content. Participants will produce their samples at home and bring it to the QIB within 24 hours of their scheduled study visit. They will also have the option of providing a sample within 24 hours after their scheduled study visit. Participants will be provided with their stool collection pack with full collection instructions (Annex 13). There will also be a AnaeroGen sachet for oxygen expulsion. Two days before the participant is due to produce their sample, participants will be requested to freeze their 3 ice-packs, so

they are frozen in good time. Participants will be requested to place their bagged stool sample between the ice-packs in order to preserve the integrity of the sample and the viability of as many microorganisms as possible until the sample is brought to QIB by the participant. The collection protocol has been developed according to International Human Microbiome Standards (IHMS) guidelines [39].

13.1.2.1 Stool Laboratory Analyses

Upon receipt at QIB, the sample will be logged, dispersed into a homogenous suspension and aliquoted (~0.5g each). Some aliquots may be used immediately upon receipt for the extraction of microbes (bacteria, viruses, fungi, archaea), for use in faecal microbiome transplantation studies, for extraction of faecal water containing metabolites and other microbial products, and/or DNA/RNA. The remaining aliquots will be stored frozen with or without the addition of preservatives (e.g. glycerol) for future ethically approved studies.

Microbiome analysis will include using high-throughput DNA/RNA sequencing methodologies (e.g. Illumina Mi/HiSeq) according to IHMS guidelines for sequencing and data analysis and specific protocols and bioinformatics pipelines established at QIB that have been developed and used in previous clinical microbiome studies. This includes:

• Optimised DNA/RNA extraction protocols, and library preparation for bacterial, viral, fungal and/or archaea species and taxonomic identification.
• A subset of samples (selected based on clinical metadata and microbial profiles) will be analysed for host (immune/metabolite) molecules to determine which factors (e.g. medications and diet) alter the host gut environment.
• Next-generation-sequencing-based analysis of faecal microbiome will be coupled with culturing methodologies (single and complex) to identify new strains of health-promoting bacteria (e.g. *Bacteroides*, *Bifidobacterium* and *Lactobacillus,* or *Ruminoccocus* species), and potential pathogens, with corresponding whole genome sequencing performed.
• Microbial analysis will be correlated with health outcomes by referring to detailed clinical information (hospital and GP notes).

13.1.3 Blood Sample

Why are we collecting these samples?

Blood contains various chemicals and proteins (biomarkers) the levels of which are used clinically to evaluate the function and health of key organs including the heart [e.g. high sensitive Troponin], liver [e.g. ALT (alanine aminotransferase)], kidneys [creatinine], bones [e.g. Calcium], and thyroid [e.g. Thyroid Stimulating Hormone]. In addition, certain gut microbes may influence the immune system which can be investigated by examining the blood for activated immune cells and their products (e.g. cytokines). Therefore, analysis of blood samples provides insights into organ and immune health of individual study participants throughout the study period and will be of value in guiding and refining further analyses of the samples.
Blood samples will be taken at the QI CRF by an appropriately trained researcher using appropriate vacutainers for harvesting PBMCs (peripheral blood mononuclear cells) and serum; 2 x 9.5mls for serum and 2 x 10mls for PBMCs. Samples will be aliquoted and then analysed immediately (e.g. for whole blood cell counts and haemoglobin concentration) or, stored at -80°C in the Norwich Biorepository prior to later use during and until the study end date.

13.1.3.1 Blood Laboratory Analyses

Whole blood will be used to determine total red cell counts, haemoglobin concentration and for assessment/quantitation of key biochemical markers of organ function and health (e.g. bone, liver, heart, kidney and thyroid). All of these have been identified as significant covariates for microbiome composition [40].
The Full Blood Count, Biochemistry and Troponin C analyses will be undertaken by NNUH Department Laboratory Medicine working to nationally agreed standards set out by Clinical Pathology Accreditation (CPA) and the Medicines and Healthcare Products Regulatory Agency (MRHA).
Serum analyses will include, for example, assays to characterise immunoglobulins and antibodies; metabolites; inflammatory mediators (e.g. cytokines); and the presence of microbes and microbial products using established protocols within QIB.

14 SAMPLE STORAGE

Samples (stool, tissue biopsies and blood) sent to QIB will be aliquoted and stored in -80°C freezers in QIB, and a second aliquot will be stored in -80°C freezers in the Norwich Biorepository. Some samples will be used fresh at QIB to enable the isolation and extraction of highly labile entities (e.g. chemicals, compounds, nucleic acids and microbes) that are rapidly degraded or lost upon removal from the body:

For example, obligate anaerobic microbes that can only survive in the anoxic, oxygen free, environment of the large intestine. In addition, the success of cell and tissue (organ) culture relies on the processing of tissue as soon as possible after procurement. Remaining samples will be aliquoted and frozen as they are, and other aliquot samples will be placed in preservation buffers (e.g. RNAlater) before storage at -80°C in the Biorepository.

14.1 Long-Term Sample Storage

After processing, all samples will be appropriately aliquoted following study-specific SOPs and will be stored in the QIB freezer facilities prior to analysis. Additionally, aliquots will be stored at the Norwich Biorepository at -80°C for back-up purposes as described above.

In terms of long-term storage after the end of the study and when data analysis is completed, the Norwich Biorepository has appropriate ethics approval which was granted by the NHS Health Research Authority (East of England-Cambridge East Research Ethics Committee) in March 2014 and samples generated from this study may be accessed by other researchers with appropriate ethical approval.
The Norwich Biorepository will have access to and hold clinical information on each participant (on secure NHS systems). The standard Biorepository (tissue bank) consent form is completed by the participant and a copy will be sent to the Biorepository. The Study Researcher will be issued with a Biorepository registration number for each sample. The Biorepository reference number will mean that the sample is anonymised. All study samples will be appropriately labelled with the Biorepository number. The Norwich Biorepository will also have access to GP notes as it has consent to access both retrospective and prospective data.

15 DATA COLLECTION

15.1 Cognitive Tests and Associated Questionnaires

Why are we collecting this data?

Cognition comprises different cognitive domains (e.g. memory, executive function, navigation) in the brain. For the current study it is important for us to determine the cognitive status of participants, as this will determine whether they are at-risk of developing dementia and which particular cognitive domains are affected. The mini-ACE will give a general overview of cognitive intactness in participants via an

objective test. This is complemented by the CCI which allows participants themselves to report whether they have any concerns in regard to their cognition. In addition to those general tests, more specialised cognitive tests, targeted towards the first changes commonly seen in dementia, will be employed to tap into specific cognitive domains. For spatial navigation, which is currently emerging as the earliest cognitive sign of dementia disease pathological processes, we will use the Supermarket and Sea Hero Quest tests. The RCFT allows a stricter assessment of episodic memory in participants, which is currently still seen as the gold standard of dementia cognitive changes. Finally, the TMT allows to detect more slowing down of response and problems switching tasks, which is commonly found in more vascular brain changes and vascular dementia. These tests will be complemented by 3 additional questionnaires, PHQ-9 allows establishment of depressive symptoms which can impact on cognitive performance, CBI-R allows establishment of any behavioural/neuropsychiatric symptoms which can impact on cognition; finally, BPQ is a questionnaire which taps into introception, i.e. how well people are aware of their bodily functions. The BPQ will be therefore important to how people are aware of their gut and has been suggested to be a cognitive surrogate marker of gut-brain interaction levels.

15.2 Questionnaires

Why are we collecting this data?

These questionnaires include a set of questions that capture information about participant demographics and behaviour which are known to contribute to individual variations in gut microbe populations. These questionnaires have been validated in a previous longitudinal sampling study and have helped in demonstrating that the composition of an individual's gut microbiome is highly personalised and reflects their lifestyle and behaviour [41]. Therefore, these questionnaires will help us in interpreting the analysis of gut microbe populations and identifying the factors (covariates) that help explain differences within and between individuals over time.

The questionnaires have been reviewed by members of the Norfolk and Suffolk Primary and Community Care Research Advisory Group that was founded by and is chaired by the CI (Professor Simon Carding). This Advisory Group is comprised of GPs, hospital consultants or scientists with an interest in gut health based at QIB or UEA. The Norfolk and Suffolk Primary and Community Care Research Office (NSPCCRO), which represents all regional clinical commissioning groups and

community care organisations also coordinated a review of the questionnaires by Patient Participation Groups (PPG) from four Norfolk GP sites; 12 members completing and returning the questionnaires. Comments were noted, and amendments were made to both questionnaires.

15.2.1 Full Health Questionnaire

Participants will be asked to provide a variety of demographic, lifestyle, behavioral and hygiene data at the initiation of the project using a questionnaire comprising approximately 50 questions. This questionnaire has been used and validated in previous longitudinal studies of the human gut microbiota and identifies key environmental factors that are known to impact on the structure and/or function of gut microbes [41]. In addition, at the June 2018 meeting of the Executive Board of the International Human Microbiome Consortium (IHMC), an international body that aims to coordinate the activities and policies of international groups studying the human microbiome, a recommendation to adopt health questionnaires for all human microbiome studies, of which the one described by Flores [41] is an exemplar, was made. Of note, the MOTION Chief Investigator and QIB are the UK representatives on the IHMC Executive Board.

15.2.2 Partial Health Questionnaire

The partial questionnaire is used to collect information about changes in health status, medication use and any other dramatic changes in the routine of the participants at the time of producing follow up samples.

15.3 Electronic Frailty Index

Why are we collecting this data?

The electronic frailty index (eFI) helps identify and predict adverse outcomes for older patients in Primary Care. The eFI is made up of 36 deficits comprising around 2,000 Read codes and is a ‘cumulative deficit’ model, which measures frailty on the basis of the accumulation of a range of deficits, which can be clinical signs (e.g. tremor), symptoms (e.g. vision problems), diseases, disabilities and abnormal test values. Many of these deficits are associated with age-associated changes in gut microbe populations. The eFI score is strongly predictive of adverse outcomes and has been validated in large international studies. Data will be requested from the participant’s GP retrospectively at the end of the study.

15.4 Physical Measurements

Why are we collecting this data?

These measurements will provide important information regarding the general health and physical fitness of individuals and identify any changes during the study period: Physical health declines during ageing which seriously compromises normal functioning in the ageing population. These measurements will also enable us to determine whether (declining) health and physical fitness is associated with any changes in cognitive function, vision and/or gut microbe populations.

To be taken at the start of the study to establish baseline values and inform on general health status and at 12-month intervals thereafter to detect any changes in health status that may be correlated with changes in other parameters being assessed and in particular, with any changes in an individuals' microbiome composition.

15.4.1 Height and weight measurement to calculate Body Max Index (BMI)

BMI, as determined by height and weight measurements, has been identified as a major covariate that accounts for variation in gut microbiome between different individuals in large population based studies [40]. BMI measurements will help assess weight stability and identify rates of gain or loss of weight over time. Excessive weight gain and obesity have been linked with changes in gut microbe populations and their metabolic activity.

15.4.2 Body composition measurements

The TANITA body composition scales use biometric impedance analysis to obtain body composition values for fat, muscle, body water and bone in addition to assessing basal metabolic rate and BMI.

15.4.3 Blood Pressure (BP)

A sphygmomanometer will be used to determine participants blood pressure as an indicator of cardiovascular health and to identify changes in cardiovascular health and hypertension throughout the study. One reading from each arm will be taken after the participant has been seated for 5 minutes.

15.4.4 Hand grip strength measurement

Both hands to be measured twice as an indication of muscle strength and any single hand dominance and how this change over the course of the study in comparison to other health status indicators.

16 EARLY TERMINATION/DISCONTINUATION/WITHDRAWAL OF PARTICIPANTS FROM STUDY

Each participant has the right to withdraw from the study at any time. In addition, the Investigator may discontinue a participant from the study at any time if the Investigator considers it necessary for any reason including:

- ❖ Cognitive test result at the Pre-Study Visit does not fall within the required threshold (Annex 26). This will also apply in the event of any cohort that already has the maximum number of 120 participants.
- ❖ Withdrawal of Consent (Annex 28).
- ❖ Ineligibility (either arising during the study or retrospectively having been overlooked at screening). See Section 7.5.
- ❖ Significant protocol deviation.
- ❖ Significant non-compliance with study requirements.
- ❖ Lost to follow up.

The reason for withdrawal will be recorded in the Study Withdrawal Log and the participant and their GP will be sent a Withdrawal Letter (Annex 27) from QIB explaining the reason for withdrawal. Participants who send their Study Withdrawal Postcard (Annex 29) will be sent a Participant Self-Withdrawal letter (Annex 28). Annexes 28 and 30 will explicitly state that the participant should contact the QIB Researchers if they wish to have any samples or data already collected to be destroyed, otherwise these will be included in the analysis.
Withdrawn participants or participants who withdraw themselves will not be replaced. In the event of Early Termination or Withdrawal, there will not be any additional visits.

17 DEFINITION OF END OF STUDY

The end of study is the date of the last visit of the last participant.

18 DEFINITION OF SERIOUS ADVERSE EVENTS

A serious adverse event is any untoward medical occurrence that:

- ❖ Results in death

- Is life-threatening
- Requires inpatient hospitalisation or prolongation of existing hospitalisation
- Results in persistent or significant disability/incapacity

Other ‘important medical events’ may also be considered serious if they jeopardise the participant or require an intervention to prevent one of the above consequences.
NOTE: The term "life-threatening" in the definition of "serious" refers to an event in which the participant was at risk of death at the time of the event; it does not refer to an event which hypothetically might have caused death if it were more severe.

18.1 Reporting Procedures for Serious Adverse Events

A serious adverse event (SAE) occurring to a participant should be reported to the REC that gave a favourable opinion of the study where in the opinion of the Chief Investigator the event was ‘related’ (resulted from administration of any of the research procedures) and ‘unexpected’ in relation to those procedures. Reports of related and unexpected SAEs should be submitted within 15 working days of the Chief Investigator becoming aware of the event, using the HRA report of serious adverse event form [42].

19 STATISTICS AND ANALYSIS

19.1 Description of Statistical Methods

A major aim of the study is the establishment of a collection of data and associated samples that will address many diverse current and future research questions regarding the role of the microbiome in ageing. It is not possible to describe all of the statistical analysis that will be conducted.

Indicative outlines of core analyses to address the main study objectives are described below. Details will vary depending on the nature of the data collected, methodological developments in the rapidly evolving area of microbiome analysis and emerging scientific literature throughout the study:

Interim/monitoring analyses

Interim reports will focus on monitoring the progress of the study against the main objectives to inform any necessary changes in protocol. These will include, as relevant at each stage of the study:

- Monitoring the rate of eligible participants from each stream,
- Participation rate of GPs
- Participation rate among different patient groups
- Rate of accruals from each stream
- Rate of accruals into each cohort
- Proportion consenting to each element of the study
- Demographic and clinical characteristics of the cohorts
- The completeness and quality of data and samples being received
- Patient's adherence to the sample collection protocols
- Response rate to sub-studies
- Attrition rate across different patient groups in each wave
- Rate of adverse events

Cross-sectional analyses (following completion of baseline assessments)

Following the completion of baseline data collection, we will have data on the composition of the gut microbiome and associated clinical, demographic and lifestyle data on all participants. This can be used to address the following questions:

1. What is the association between age and the composition of the gut microbiome, and what is the variation in gut microbiome composition and function among middle aged and older people in Norfolk?
2. How do differences in lifestyle between age-groups account for these differences?
3. Is the microbiome associated with cognitive function and associated symptoms, physical function or other markers of successful ageing?
4. Can age or lifestyle differences account for any associations identified in 3 above, or
5. Does the gut microbiome mediate the effects of age and lifestyle on health?

To address each question, both univariate and multivariate analyses will be used. For questions 1-2, characteristics of the microbiome would be considered as the outcome (dependent) variable and age and other demographic factors will be

entered as predictor (independent) variables. For questions 3-5, the clinical outcome measures would be considered the outcome variables with microbiome characteristics, demographic and lifestyle data being considered as predictors. Mediation analysis will estimate to what extent composition of the microbiome can account for the effects of age and lifestyle on each outcome.

Longitudinal analyses (after successive waves and following completion of all data collection)

Detailed analyses plans will be prepared following data collection but ahead of any analysis after each visit, building on the cross-sectional findings, but in brief planned analyses will address the following questions:

6. What is the intra-individual variation and trajectory of different components of the gut microbiome with age?
7. How does the baseline composition of the gut microbiome predict future changes in clinical outcomes? Eg, among people with mild cognitive impairment, can analysis of the gut microbiome be used to predict future cognitive change.
8. How do changes in the gut microbiome correlate with changes in clinical outcomes?

Multiple testing and mitigating the effects of false positives

Throughout, careful attention will be paid to the possibility of false positive results occurring through multiplicity given the large number of hypotheses being tested and the large number of parameters to be set in in bioinformatics and statistical techniques. This will be mitigated as far as possible for each hypothesis by clearly pre-specifying each individual analysis in a statistical analysis plan detailing the coding of exposures, outcomes and covariates and primary statistical methods to be employed including any subgroup analyses. These statistical analysis plans will be developed and will be pre-registered following data collection so that the distributions of key variables are known but before relevant analysis linking microbiome and health outcome information is undertaken. Exploratory and secondary analyses will be clearly reported as such in all outputs, will use accepted techniques to cross-validate findings and correct significance tests for multiplicity, and results from all analyses will be published irrespective of whether findings are predominantly positive or negative.

19.2 The Number of Participants

The study will recruit 360 participants. Over four years we anticipate that roughly 20% of participants will die or drop out of the study, reflecting rates seen in national cohort studies in similar populations over similar periods of time, hence 300 participants would be available for analysis at the end of the study.

A primary aim of this study is to estimate the link between microbiome composition and longitudinal change in clinical outcomes. A simulation study was conducted to estimate power for this objective, using as inputs the dynamics of cognitive change estimated from The Irish Longitudinal Study on Ageing [43]. There are many unknowns with respect to the prevalence of the microbiome features that might be associated with cognitive decline, the size of their effects, and variation in outcome measures. There is no prior data on our primary cognitive outcome measure (Mini-ACE), and so a generic measure of cognitive function (Mini-Mental State Examination, MMSE) was used, which is likely to have similar measurement properties. The standard deviation in MMSE change over four years was 2.2 points in a dataset resampled from the TILDA study [43] as per the MOTION protocol. The smallest clinically meaningful difference in MMSE score has been reported as 1.4 [44], although much smaller effects are likely to be of scientific or public health importance. The power to detect effects of 1.5, 1.0, 0.8 and 0.5 MMSE points (corresponding to standardised effect sizes of 0.68 standard deviations to 0.22 standard deviations) over 4 years using a mixed effects model with five assessments were estimated by simulation (at $p<0.05$), assuming prevalence of the specific microbial feature causing the effect is between 5% and 50%. These are shown below, showing that under reasonable assumptions the Motion study will have enough power to detect moderate effects of relatively prevalent features, and large effects of rare features.

The simulation was conducted using the simr package in R statistical software [44].

Power (%)	Prevalence (%)				
**Effect (MMSE points; standardised effect size)**	50	25	10	7	5
**0.5; 0.22**	50	39	22	-	-
**0.8; 0.36**	90	**77**	47	-	-
**1; 0.45**	99	97	67	-	-
**1.5; 0.68**	-	-	99	**87**	50

20 STUDY MANAGEMENT PLAN

20.1 Trial Management Group (Study team members)

The Trial Management Group including the Chief Investigator (Professor Simon Carding), Gut Microbes and Health QIB Clinical Studies Officer (Shelina Rajan), two Microbiome Technicians, NHS Principal Investigators and CRN Research Nurses will be responsible for the day-to-day management of the trial. They will monitor all aspects of the conduct and progress of the trial, ensure that the protocol is adhered to and take appropriate action to safeguard participants and the quality of the trial itself. A delegation log will be used for recording the roles and responsibilities of the local research team and the authorisation of the Principal Investigator (PI) for this (Staff signature and delegation log, HRA Template version 4.2, 2nd January 2018). This log will include the main study activities that the PI can delegate to staff at the participating organisation. The task list and delegation log will be maintained as an up to date document throughout the duration of the study at the participating organisation.

20.2 Trial Management Oversight Group (Non- study team members)

Management of the study will be overseen by an internal QIB Study Core Management Team who are not directly involved in any aspect of study conduct. The team will consist of the following representatives: -

- Health, Safety, Environment and Quality Assurance.
- QIB Sponsor Representative.

- ❖ Gut Microbes and Health QIB Programme Manager.
- ❖ QIB Statistician.
- ❖ QIB Bioinformatician.
- ❖ Information Technology Security Specialist.
- ❖ Norwich Biorepository.
- ❖ Patient and Public Involvement.

The Core Trial Management Group will be responsible for overseeing the running of the study. They will ensure the monitoring of and facilitating the progress of the study, and ensure the study is delivered within the projected timelines is achieved using key performance indicators. Recruitment targets, success of data collection, and any specific issues arising will be addressed.

The role, constitution and composition of the Trial Management Oversight Group will be fully described in a separate document that will be kept within the Trial Master File. Briefly, the Chief Investigator and Sponsor will appoint the Chair and members. The primary reporting line of this group is via the Chair to the QIB Human Research Governance Committee; however, communication is likely to be between the Chair and the Study Manager (Gut Microbes and Health QIB Clinical Studies Officer (Shelina Rajan) who has day to day responsibility for the project. Although there may be periods when more frequent meetings are necessary, the Trial Management Oversight Group will meet at least every 6 months. Minutes of meetings will be sent to all members and the sponsor and be retained in the Trail Master File. The Chief Investigator, in association with the Chair, will be responsible for calling and organising meetings.

21 DATA MANAGEMENT PLAN

21.1 Description of the data

Our data collected for this study will be the following: -

❖ Cognitive Assessments and associated tests and questionnaires.

❖ Health and Lifestyle Questionnaires (general information, dietary intake, allergies/intolerances, general lifestyle and hygiene, general health, medical conditions and current medication/antibiotic use.

❖ Physical Measurement Collection (height, Tanita Body Composition measurements, blood pressure and hand grip strength measurements).

- ❖ Biological Sample data (colonoscopy tissue biopsies from subgroup 1 and FBC, Biochemistry and Troponin C results will be collected from NNUH ICE system) by NHS staff working with the Study Research Team. Additional blood samples (Section 13.1.3) and stool samples will undergo analysis at QIB.
- ❖ Clinical Test data (brain MRI scans - subgroup 2 and OCT (A) scans).
- ❖ Other data (eFI data collected retrospectively from participant's GP).

All of the above collected data will be anonymised and stored on a secure IT (Information Technology) system which only the Study Researchers will have access to.

21.2 Specific management of personal data

Research participants who will be successfully recruited onto the study will be assigned a unique code number which will be kept in a secure file. A lockable filing cabinet or cupboard will be used to keep paper documents that include the file linking the participant to the code and personal information. Manual files/folders will consist of separate named and numbered files for each participant. No data with the participants' name will be filed in the numbered file and vice versa. Only the study scientists will have access to the file linking personal data to the participants' unique code. Confidential data will be accessed only by the study team at the investigator site. All electronic data will be stored on a password protected shared data file. Only the study team will have access to these data. Data will be stored for at least 15 years after completion or discontinuation of the study in a confidential archive. Archived data will not be used to contact participants after the end of the study. Access to archived data will be limited to the study scientist and chief investigator (CI) of the study or the CI's successor. The quality assurance auditors may also be allowed access with the permission, and in the presence, of the CI. Any information collected prior to consent via study-specific advertising material will be used solely for the purpose of the study and will be handled in compliance with the QIB Data Protection Policy in order to protect and respect subjects' privacy. This process has reviewed and authorised by QIB Data Protection Officer. Data will be managed by the study team in compliance with EU General Data Protection Regulation (GPDR) and UK Data Protection Act 2018.

21.3 Specific management of samples:

All biological samples collected as part of the study will be known only by their unique code number. All data collected will also be identified by code only.

Laboratory results will be maintained in a spreadsheet form and will be in file formats that can be shared internally. Only anonymised individual-level data will be shared within study team members (QIB and NHS investigators).

21.4 Data collection/generation

Data from Section 21.1 will be collected by trained researchers onto study-specific forms and then uploaded into electronic data sets. Data from secure NHS IT systems will be collected by authorised Study Researchers completing case report forms (CRFs) and will be used to build encrypted databases. Methods used to generate data from this study will be fully described in standard operating procedures (SOPs). The study record will include a detailed description of data collection and coding system.

All raw data will be collected on a continuous basis and supplemented with relevant additional information (e.g, identity of researcher collecting and entering data, date of collection). Most of the data will be in digital form; however, some data will originally be recorded in hardcopy-form and later transcribed to digital copies. Working copies of all datasets will be kept in an encrypted format on the institutional network (QIB) which has shared access with appropriately authorised research staff working on the project.

We will ensure that clear audit trails link secondary processed information to primary data and will be audited in compliance with International Good Clinical Practice (GCP) standards.

21.5 Data sharing and access

The research protocols will be registered in a publicly accessible database after gaining favourable ethical opinion. Registration to ClinicalTrials.gov Protocol Registration and Results System (PRS) using QIB account will allow us to be transparent in our work. The study team will ensure fully compliance with the standards required for deposition of information in any relevant public databases. Anonymised datasets will be kept indefinitely and available to other researchers to comply with journal requirements when required. Study results will also be submitted in Clinicaltrials.gov and made available in a timely manner. Summary results will be submitted as four separate modules (Participant Flow, Baseline Characteristics, Outcome Measures and Statistical Analyses and Adverse Events) in compliance with the requirements of submitting results of ClinicalTrials.gov [45].

Consent forms clearly state the data sharing procedures for data generated from this study.

21.6 Relevant institutional policies on data sharing and data security

All data will be managed, protected and shared in accordance with the requirements of the QIB Quality Code of Practice, the QIB Policy on Safeguarding Good Scientific Practice and the BBSRC Data Sharing Policy. All study collaborators will adhere to the same rigorous standards for data management.

Direct access will be granted to authorised representatives from the Sponsor and host institution for monitoring and/or audit of the study to ensure compliance with regulations.

Direct access will be granted to authorised representatives from the Sponsor and host institution for monitoring and/or audit of the study to ensure compliance with regulations.

21.7 Data Recording and Record Keeping

Data will be managed by the study team in compliance with EU General Data Protection Regulation (GDPR) and UK Data Protection Act 2018.

21.7.1 Data Retrieval & Storage

Members of NHS staff who are part of the research team will access the NNUH ICE system where clinical results will be securely held. Where practicable, the Study Researcher will transfer the anonymised study-related data on to the secure IT system and access to this system will be limited to only those authorised on the Study Team.

Where it is not practicable to transfer data, for example quite detailed brain MRI scan reports, these will be printed, anonymised and the participants study ID number will be applied. These will be stored in a locked cabinet with access restricted.

22 QUALITY ASSURANCE PROCEDURES

The study may be monitored, or audited in accordance with the current approved protocol, GCP, relevant regulations and SOPs.

23 ETHICAL AND REGULATORY CONSIDERATIONS

23.1 Declaration of Helsinki

The Investigator will ensure that this study is conducted in accordance with the principles of the Declaration of Helsinki. The proposed research will be conducted in accordance with the conditions and principles of the International Conference on Harmonisation GCP, and in compliance with national law. The research will meet the requirements of the new EU General Data Protection Regulation (GDPR), UK Data Protection Act 2018 and relevant sponsor's policies.

23.2 Approvals

The protocol, informed consent form, participant information sheet and any proposed advertising material will be submitted to an appropriate Research Ethics Committee (REC), and HRA for written approval. The study protocol and associated documents will be reviewed by the Human Research Governance Committee (HRGC) at QIB and approved by the QIB statistician prior to submission to the REC. QIB HRGC adheres to the UK Policy Framework for Health and Social Care Research. Copy of the HRGC approval, and any correspondence with the committee will be available to the REC and HRA, if requested.

The Investigator will submit and, where necessary, obtain approval from the above parties for all substantial amendments to the original approved documents.

23.3 Reporting

The CI shall submit once a year throughout the study, or on request, an Annual Progress report to the REC Committee, HRA (where required) host organisation and Sponsor. In addition, an End of Study notification and final report will be submitted to the same parties.

23.4 Participant Confidentiality

The study staff will ensure that the participants' anonymity is maintained. The participants will be identified only by a participant ID number on all study documents and any electronic database, except for the Consent Form, where participant name and initials will be added by the participant. All documents will be stored securely and only accessible by study staff and authorised personnel. Volunteers who are successfully recruited onto the study will be assigned a unique code number which will be kept in a secure file. A lockable filing cabinet or cupboard will be used to keep paper documents that include the file linking the participant to

the code and personal information. All electronic data will be stored on a password protected shared data file. Confidential data will be accessed only by the study scientists. The samples sent to the other laboratory facilities will be known only by their code number. All data collected will also be identified by code only.

Participants' personal data will be held in a locked cabinet or password protected electronic file in QIB. Only the study scientists will have access to these data. Data will be stored for at least 15 years after completion or discontinuation of the study. The data will be stored in the QIB human studies archive. Archived data will not be used to contact participants after the end of the study. Access to archived data will be limited to the study scientist and chief investigators (CI) of the study or the CI's successor. The quality assurance auditors may also be allowed access with the permission, and in the presence of the CI.

The main computer storage will be on one main QIB computer, but as part of a password protected shared network. All QIB computers are individually password protected and the shared network access is limited to those working within the research area. Only the study scientists will have access to the file linking personal data to the participants' unique code. Manual files/folders will consist of separate named and numbered files for each participant. No data with the participants' name will be filed in the numbered file and vice versa.

The study will comply with EU General Data Protection Regulation (GDPR) which came into force in the UK on 25 May 2018 and the UK Data Protection Act 2018, which requires data to be anonymised as soon as it is practical to do so. Personal data will be subjected to appropriate safeguards and a specific SOP will be established with the QIB Data Protection officer. This will ensure that the study team will have appropriate organisational and technical measures in place to ensure that data are processed lawfully, fairly and in a transparent manner and are kept to a minimum and secure in the research context.

23.4.1 Use of audio/video recordings and anonymised quotes

During participation in the study, the participant may receive a letter from the Study Researcher (Annex 37) inviting them to attend the QI CRF to provide an audio/video recording. This material will be used to gather their experience of taking part in the study, to promote public awareness of taking part in human studies and will therefore serve to inform the design of longer scale studies in the future. The Study Researcher will always respect the participant's right to privacy and anonymity where expressed. The participant may also be asked to provide anonymised quotes for use in future research reports and publications. The participant will be made

aware that they can decline this invitation and that it would not affect their participation in the study.

23.5 Participant Identifiable Data Received Electronically

For participants who reply online (www.quadram.ac.uk/motionstudy) or via e-mail (motion@quadram.ac.uk), identifiable data will be received by only those Study Researchers authorised to do so.

The completed online form (Screen shot Annex 7.1) will be delivered directly to the MOTION Study inbox (motion@quadram.ac.uk) which is the same e-mail address participants would use to e-mail the Study Researcher directly. The Privacy Notice (Screen shot Annex 7.2) details what the participant would read about detailing what will happen to their data. The participant will see the link to the Privacy Notice before they click on the submit button. The online form and the Privacy Notice have been approved by the QIB Data Protection Officer (Annex 7.3).
Any identifiable data received on any other electronic device will be deleted.

23.6 Expenses and Benefits

Reasonable travel expenses for all study-related visits will be paid. Mileage will be paid at the current QIB standard rate of 45p per mile. Taxi or bus fares will be reimbursed on production of a valid receipt. Car parking charges will also be reimbursed.
The only exception to this is the date of the colonoscopy as the participant would be attending the QIE Unit as part of routine clinical care.
As a thank you to participants and to compensate for any inconvenience, each participant will receive a Love2shop voucher worth £10 at Study Visit 4 and a further voucher to the value £15 upon study completion at Study Visit 8.

23.7 Other Ethical Considerations

23.7.1 Vulnerable Participants

Any participants who the researcher felt would not be able to consent for themselves or comply with or understand the study requirements would not be recruited in to the study.
Where it is felt that a participant has/is losing capacity to continue in the study, they will be sensitively withdrawn from the study as stated in Section 16.

24 FINANCE AND INSURANCE

24.1 Funding

The study has been funded by Biotechnology and Biological Sciences Research Council (BBSRC) through an Institute Strategic Programme (ISP) award to the QIB Gut Microbes & Health Programme. It is envisaged that this study will be adopted into the NIHR portfolio which will provide additional support in terms of hospital infrastructure and staff support. Due to the nature of funding, this study will be considered eligible subject to the sponsor confirming that peer review is undertaken in line with the eligibility criteria.

24.2 Insurance

QIB has £10 million commercial insurance for human trial studies/clinical trials, including no fault liability insurance. The study will be covered by the current QIB Insurance (Annex 30) and the Letter of Study Indemnity (Annex 32) which gives further details of QIB liability insurance.

NHS bodies are legally liable for the negligent acts and omissions of their employees. If any participant is harmed whilst taking part in a clinical research study as a result of negligence on the part of a member of the study team, this liability cover would apply.

Non-negligent harm is not covered by the NHS indemnity scheme.

25 PUBLICATION POLICY

The study will be registered in a publicly-accessible database after gaining favourable ethical opinion. Registration to ClinicalTrials.gov Protocol Registration and Results System (PRS) using the QIB account will allow us to be transparent in our work and meet legal requirements. The Investigators will be involved in reviewing drafts of the manuscripts, abstracts, press releases and any other publications arising from the study. Authors will acknowledge that the study was funded by BBSRC. Authorship will be determined in accordance with the ICMJE guidelines and other contributors will be acknowledged. All publications will be open access as per BBSRC guidelines.

26 CORE TEAM MEMBERS AND EXPERTISE

Professor Simon Carding is Head of the Gut Microbes and Health programme at QIB and Professor of Mucosal Immunology at the Norwich Medical School (UEA). He has

expertise in gut biology, microbiology and immunology and has past experience of managing large multidisciplinary basic and applied biomedical research projects involving multiple partners and research groups within QI and in universities in the UK and Europe. He is also the founder of the Norfolk and Suffolk Primary and Community Care Research Advisory Group that aims to foster and develop research involving patients in primary and community care.

Dr Simon Rushbrook is an NNUH consultant in Gastroenterology and Hepatology and is Clinical Director of NNUH Endoscopy. His clinical expertise includes the management of patients with acute and chronic liver disease and chronic biliary tract disease. His research interests are the genomics of liver disease for which he has established a UK consortium to collect DNA samples from patients with Primary Sclerosing Cholangitis.

Professor Michael Hornberger has expertise in Cognitive Science, Neurology and Neuropsychology. His research focuses on improving diagnosis, disease progression tracking and symptom management in Dementia.

Dr Ben Burton is a Consultant Ophthalmologist with particular expertise in cataract surgery, age related macular degeneration, diabetic retinopathy, uveitis and neuro-ophthalmology. He has pioneered research in the James Paget Hospital eye department, and it is now the most research active department in the hospital. He was appointed Clinical Research and Development Director for the trust in 2015.

Mrs Shelina Rajan is the Clinical Studies Officer at QIB. She has over 15 years research experience as a Senior Research Nurse in participant recruitment in Industry and in both Primary and Secondary Care. This includes taking informed consent, study set-up and delivery to time and to target.

Dr Judy Henwood is Research Design Lead for the Norfolk and Suffolk Primary and Community Care Research Office. Following a PhD in Molecular Immunology and a series of personal Fellowships to fund a career in laboratory-based research, she joined the Research Office in 2009. Leading the design and development arm of the Research Office on behalf of the Clinical Commissioning Groups, her role is to work at the interface between academics and clinicians to ensure the development of robust research programmes that are clinically relevant. She will provide an enduring link with the Research Office, community care, general practice and the CCGs which will support the recruitment and retention of participants in the study.

27 Schedule of Events (Annex A)

	PHASE 1 Study Schedule		Phase 2 Study Schedule								
	**Telephone Contact**	**Pre-Study Visit**	**Baseline Visit**	**Study Visit 1**	**Study Visit 2**	**Study Visit 3**	**Study Visit 4**	**Study Visit 5**	**Study Visit 6**	**Study Visit 7**	**Study Visit 8**
			**Up to 3 weeks from Pre-Study Visit**	**6m +/- 1 month**	**12m +/- 1 month**	**18m +/- 1 month**	**24m +/- 1 month**	**30m +/- 1 month**	**36m +/- 1 month**	**42m +/- 1 month**	**48m +/- 1 month**
Telephone Call	X										
Informed Consent		X*									
Cognitive Assessment		X[1]*	X[2]*	X[2]*		X[2]*		X[2]*		X[2]*	
Pick-up stool collection pack/items		X*	X*	X*	X*	X*	X*	X*	X*	X*	
Stool sample drop-off			X*	X*	X*	X*	X*	X*	X*	X*	X*
Blood Sample			X*[3X]	X*[3Y]	X*[3X]	X*[3Y]	X*[3X]	X*[3Y]	X*[3X]	X*[3Y]	X*[3X]
Optical Coherence Tomography (OCT) [4] Scan			X[a] Within 4 weeks from Baseline				X				X[a] Within 4 weeks prior to Study Visit 8
Health Questionnaire			X[5]*	X[6]*	X[6]*	X[6]*	X[6]*	X[6]*	X[6]*	X[6]*	X[6]*
Brain MRI Scan [7]			X within 6 weeks from Baseline								X within 6 weeks before Study Visit 8
Physical Measurement Collection [8]			X*		X*		X*		X*		X*
Electronic Frailty Index Data Collection from GP											X[9]
Colonoscopy Tissue Biopsy [10]			Stream 1 Endoscopy at any time point during the study<br>Stream 2 Endoscopy between Pre-Study Visit and Study Collection (Baseline)<br>Stream 3 Endoscopy at any time point during the study								**Collection at 2 time points where possible**

* QI CRF (Quadram Institute Clinical Research Facility).

[1] mini ACE(Addenbrooke's Cognitive Examination) and CCI (Cognitive Change Index) are used to determine final eligibility

[2] mini ACE, CCI, PHQ9, Supermarket Test, Sea Hero Test, CBI-R (Cambridge Behavioural Inventory-Revised), RCFT (Rey Complex Figure Test), TMT (Trail Making Test) and BPQ (Body Perception Questionnaire) and PHQ9 (Patient Health Questionnaire) The CCI, PHQ9, CBI and BPQ are self-administered tests.

[3X] Blood for serum and plasma

[3Y] Biochemistry Troponin C and Full Blood Count

[a] Stream 2 receive OCT (Angiography) scans

[4] Beccles and District War Memorial Hospital

[5] Full Health Questionnaire

[6] Partial Health Questionnaire

[7] NNUH (Norfolk & Norwich University Hospital) n=30 from Cohort 3

[8] Height, weight, BP, Tanita scale & hand grip strength measurements

[9] Provided by participant's GP

[10] QI Endoscopy Unit NNUH n = 90 (30 from each cohort)

28 REFERENCES

1. Carding, S., et al., *Dysbiosis of the gut microbiota in disease.* Microb Ecol Health Dis, 2015. **26**: p. 26191.
2. Korpela, K., et al., *Childhood BMI in relation to microbiota in infancy and lifetime antibiotic use.* Microbiome, 2017. **5**(1): p. 26.
3. WHO, *World report on ageing and health*. 2015, WHO.
4. Calder, P.C., et al., *A holistic approach to healthy ageing: how can people live longer, healthier lives?* J Hum Nutr Diet, 2018.
5. Zapata, H.J. and V.J. Quagliarello, *The microbiota and microbiome in aging: potential implications in health and age-related diseases.* J Am Geriatr Soc, 2015. **63**(4): p. 776-81.
6. Carding, S. and L. Hoyles, *The human virome.* Alimentary Pharmacology and Therapeutics, 2017. **In Press**.
7. WHO, *mental health and older adults*. 2017, WHO.
8. Prince, M., et al., *Recent global trends in the prevalence and incidence of dementia, and survival with dementia.* Alzheimers Res Ther, 2016. **8**(1): p. 23.
9. Gauthier, S., et al., *Mild cognitive impairment.* Lancet, 2006. **367**(9518): p. 1262-70.
10. Claesson, M.J., et al., *Gut microbiota composition correlates with diet and health in the elderly.* Nature, 2012. **488**(7410): p. 178-84.
11. Brussow, H., *Microbiota and healthy ageing: observational and nutritional intervention studies.* Microb Biotechnol, 2013. **6**(4): p. 326-34.
12. Ghaisas, S., J. Maher, and A. Kanthasamy, *Gut microbiome in health and disease: Linking the microbiome-gut-brain axis and environmental factors in the pathogenesis of systemic and neurodegenerative diseases.* Pharmacol Ther, 2016. **158**: p. 52-62.
13. Bedarf, J.R., et al., *Functional implications of microbial and viral gut metagenome changes in early stage L-DOPA-naive Parkinson's disease patients.* Genome Med, 2017. **9**(1): p. 39.
14. Chen, C.H., C.L. Lin, and C.H. Kao, *Irritable Bowel Syndrome Is Associated with an Increased Risk of Dementia: A Nationwide Population-Based Study.* PLoS One, 2016. **11**(1): p. e0144589.
15. Sampson, T.R., et al., *Gut Microbiota Regulate Motor Deficits and Neuroinflammation in a Model of Parkinson's Disease.* Cell, 2016. **167**(6): p. 1469-1480 e12.
16. Yang, Q., et al., *Automated layer segmentation of macular OCT images using dual-scale gradient information.* Opt Express, 2010. **18**(20): p. 21293-307.
17. Ko, F., et al., *Association of Retinal Nerve Fiber Layer Thinning With Current and Future Cognitive Decline: A Study Using Optical Coherence Tomography.* JAMA Neurol, 2018.
18. Statistics, O.N., *Estimates of the very old:2002 to 2016*. 2016.
19. Association, B.M., *Healthy Ageing: Growing older in the UK*. 2018.

20. Petersen, R.C., et al., *Mild cognitive impairment: a concept in evolution.* J Intern Med, 2014. **275**(3): p. 214-28.
21. Sachdev, P.S., et al., *The Prevalence of Mild Cognitive Impairment in Diverse Geographical and Ethnocultural Regions: The COSMIC Collaboration.* PLoS One, 2015. **10**(11): p. e0142388.
22. Sachdev, P.S., et al., *Factors predicting reversion from mild cognitive impairment to normal cognitive functioning: a population-based study.* PLoS One, 2013. **8**(3): p. e59649.
23. PHE. *NHS Bowel Cancer Screening Programme*. 2015; Available from: https://www.gov.uk/guidance/bowel-cancer-screening-programme-overview.
24. Research, J.D. 2018; Available from: http://nspccro.nihr.ac.uk/for-the-public-patients-and-carers/get-involved-in-research/join-dementia-research.
25. Magazine, N.S.F.T.I. 2018; Available from: http://www.nsft.nhs.uk/About-us/Pages/Insight-magazine.aspx.
26. Norfolk, H. 2018; Available from: https://www.healthwatchnorfolk.co.uk/about-us/.
27. Hsieh, S., et al., *Validation of the Addenbrooke's Cognitive Examination III in frontotemporal dementia and Alzheimer's disease.* Dement Geriatr Cogn Disord, 2013. **36**(3-4): p. 242-50.
28. Rattanabannakit, C., et al., *The Cognitive Change Index as a Measure of Self and Informant Perception of Cognitive Decline: Relation to Neuropsychological Tests.* J Alzheimers Dis, 2016. **51**(4): p. 1145-55.
29. Lowe, B., et al., *Monitoring depression treatment outcomes with the patient health questionnaire-9.* Med Care, 2004. **42**(12): p. 1194-201.
30. Tu, S., et al., *Lost in spatial translation - A novel tool to objectively assess spatial disorientation in Alzheimer's disease and frontotemporal dementia.* Cortex, 2015. **67**: p. 83-94.
31. Wedderburn, C., et al., *The utility of the Cambridge Behavioural Inventory in neurodegenerative disease.* J Neurol Neurosurg Psychiatry, 2008. **79**(5): p. 500-3.
32. Porges, S. *Body Perception Questionnaire*. 1993.
33. Meyers, J.E. and K.R. Meyers, *The Rey Complex Figure Test and Recognition Trial (RCTF)*. 1995: PAR Inc.
34. Reitan, R.M., *Trial Making Test: Manual for Administration and Scoring*. 1992: Reitan Neuropsychology Laboratory.
35. Kim, B.J., J. Kim, and D.J. Irwin, *The Potential Role of Optical Coherence Tomography for Dementia Patients.* Retinal Physician, 2018. **15**: p. 49-51.
36. Van Stavern, G., *Microvascular changes in the retina detected by using noninvasive optical coherence tomography (OCT) angiography align well with imaging and biomarker evidence of preclinical Alzheimer's disease*, in *American Academy of Neurology*. 2018: Los Angeles, USA.
37. Zinkernagel, M.S., et al., *Association of the Intestinal Microbiome with the Development of Neovascular Age-Related Macular Degeneration.* Sci Rep, 2017. **7**: p. 40826.

38. Bulut, M., et al., *Evaluation of optical coherence tomography angiographic findings in Alzheimer's type dementia.* Br J Ophthalmol, 2018. **102**(2): p. 233-237.
39. Standards, I.H.M. 2015; Available from: http://www.microbiome-standards.org/.
40. Falony, G., et al., *Population-level analysis of gut microbiome variation.* Science, 2016. **352**(6285): p. 560-4.
41. Flores, G.E., et al., *Temporal variability is a personalized feature of the human microbiome.* Genome Biol, 2014. **15**(12): p. 531.
42. Authority, H.R. 2018; Available from: https://www.hra.nhs.uk/approvals-amendments/managing-your-approval/safety-reporting/.
43. Dublin, T.C. *The Irish Longitudinal Study on Ageing*. 2018; Available from: http://tilda.tcd.ie/.
44. Howard, R., et al., *Determining the minimum clinically important differences for outcomes in the DOMINO trial.* Int J Geriatr Psychiatry, 2011. **26**(8): p. 812-7.
45. Tse T, W.R., Zarin DA, *Reporting "basic results" in ClinicalTrials.gov.* Chest, 2009. **136**(1): p. 295-303.
