# Supplementary material for: A protocol paper for the MOTION Study—A longitudinal study in a cohort aged 60 years and older to obtain mechanistic knowledge of the role of the gut microbiome during normal healthy ageing in order to develop strategies that will improve lifelong health and wellbeing
Source: PLoS One. 2022 Nov 18;17(11):e0276118. doi: 10.1371/journal.pone.0276118 (PMC9674124; doi:10.1371/journal.pone.0276118)
Supplement: Attachment — Submitted filename: Response to reviewers - V2 19th August 2022.docx [file pone.0276118.s003.docx]

We would like to thank the reviewers for their careful and considered review of our manuscript. Below we have provided a point-by-point response, in blue text, to the queries raised in addition to a PDF copy as well as a Word version of the document with tracked changes.

**Review Comments to the Author**

Reviewer #1: The authors defend the mixed model approach in their responses as well as the use of random and fixed terms.

However, this reviewer does not understand the comment ,"We are yet to prepare statistical analysis plans to address the main questions underlying the report, the study protocol does not specify any particular analysis and methods to statistically analyse microbiome data are ongoing rapid development, but we will carefully consider ‘epidemiological’ concerns and potential approaches such as the use of propensity scores when we are developing these. According to our study protocol analysis plans will be developed and pre-registered prior to analysis. "

Section 3.8 and Table 2 appear to address this concern. This is confusing.

**We apologise for the confusion. The analysis section of our paper outlines a number of research questions that will be answered as opposed to the specific statistical methods to address them. For the sake of a power calculation, we used a very simplified analysis linking a single binary microbiome feature (for example the baseline presence of a particular taxon) to cognitive change, but analysis of many features of the microbiome are more difficult to analyse than this being compositional, multivariate, sparse, based on counts etc. Methods to measure microbiomes and to analyse microbiome data are evolving, particularly with respect to more complex analysis (e.g., for longitudinal data) and so we have not yet finalised the statistical analysis methods for these.**

Reviewer #3: The authors have addressed all initial concerns and have adequately revised their manuscript. I have no further comments.
